# Colourimetric analysis of thermally altered human bone samples

**DOI:** 10.1038/s41598-019-45420-8

**Published:** 2019-06-20

**Authors:** Tristan Krap, Jan M. Ruijter, Kevin Nota, Joyce Karel, A. Lieke Burgers, Maurice C. G. Aalders, Roelof-Jan Oostra, Wilma Duijst

**Affiliations:** 1 Maastricht University, Maastricht, The Netherlands; 2 Amsterdam UMC, Location AMC, department of Medical Biology, Section Anatomy, Amsterdam, The Netherlands; 3 Ars Cogniscendi Foundation for Legal and Forensic Medicine, Wezep, The Netherlands; 4 Department of Life Sciences and Technology–Biotechnology–Forensic Science, Van Hall Larenstein, University of Applied Sciences, Leeuwarden, The Netherlands; 5 Amsterdam UMC, Location AMC, department of Biomedical Engineering and Physics, Amsterdam, The Netherlands; 6 Co van Ledden Hulsebosch Center, Amsterdam, The Netherlands

**Keywords:** Imaging, Biophysical methods

## Abstract

At this moment, no method is available to objectively estimate the temperature to which skeletal remains have been exposed during a fire. Estimating this temperature can provide crucial information in a legal investigation. Exposure of bone to heat results in observable and measurable changes, including a change in colour. To determine the exposure temperature of experimental bone samples, heat related changes in colour were systemically studied by means of image analysis. In total 1138 samples of fresh human long bone diaphysis and epiphysis, varying in size, were subjected to heat ranging from room temperature to 900 °C for various durations and in different media. The samples were scanned with a calibrated flatbed scanner and photographed with a Digital Single Lens Reflex camera. Red, Green, Blue values and Lightness, A-, and B-coordinates were collected for statistical analysis. Cluster analysis showed that discriminating thresholds for Lightness and B-coordinate could be defined and used to construct a model of decision rules. This model enables the user to differentiate between seven different temperature clusters with relatively high precision and accuracy. The proposed decision model provides an objective, robust and non-destructive method for estimating the exposure temperature of heated bone samples.

## Introduction

Fire is one of the most destructive natural forces. During fire, both flaming and smouldering combustion give rise to excessive heat release by radiance and convection. As a result, a thermal gradient forms with higher temperatures near the site of combustion. Due to convection, differences in density, and entrainment, hot gases will accumulate above the seat of the fire^1,2^. During a house or car fire, temperatures in the range from 700 °C to 900 °C and higher, can be reached^3–6^. Such fires are fatal and destructive to the human body^7,8^. The degree of destruction of the body depends on the temperature and duration of the fire. Excessive heat dehydrates soft tissues resulting in splitting of the skin and contraction of muscles. Dehydrated skin, muscle, and underlying adipose tissue are combustible, and will start to pyrolyze and carbonize when exposed to sufficient heat. If the fire lasts long enough the most superficial skeletal elements will be uncovered and, as the burning process continues, deeper lying skeletal elements will be exposed until the anatomical regions that are farthest away from the heat source are no longer shielded^7,9^. However, skeletal elements are in most cases relatively well preserved after a fire and are rarely completely fragmented thus making it rare to encounter merely ashes^10–12^. Therefore, bones are an important source of information. Based on heat-induced (HI) changes it is possible to reconstruct the (pre-)burning conditions, like the position of the body in relation to the heat source^7,9^. Such a reconstruction can provide key information for both forensic investigations and for archaeological analysis^9,13^. Apart from a reconstruction, it is of importance to assess the probability of obtaining a DNA- or isotope-profile from thermally altered human skeletal remains^14,15^. In both cases, evidence based information on the degree of HI changes is required.

As soon as bone is exposed to heat it will start to change. The effect of heat on bone has previously been reported to dependent on temperature, duration of exposure, available oxygen and the surrounding medium^16–20^. When exposed to a temperature exceeding 700 °C in an oxygen-rich environment, bone will roughly go through four stages: dehydration, decomposition, inversion, and fusion^21^. Due to these changes HI fractures can occur and bone will undergo observable and measurable changes in dimensions, weight, porosity, crystallinity and colour^22^. HI changes in colour are related to changes in the chemical composition of the organic components. In general, the colour changes from ivory white to brownish-black, from black into grey and finally the bone becomes pure white. These changes are mainly caused by thermal decomposition of type-1 collagen and subsequent burning away of the residual carbon^14,21–29^. In addition to the described colour gradient also minor local colour tints have been observed; the reported bluish, greenish or pinkish tints reflect incomplete combustion or the presence of metals during heating^27,30^.

Several authors have suggested the use of colour to estimate the temperature that skeletal remains have been exposed to. In general, the methods that can be deployed to analyse colour, can be divided into subjective and objective methods. Subjective methods to estimate the exposure temperature include visual colour assessment based on descriptions and comparisons with colour charts like the Munsell Colour Chart or the colour chart for heated bone provided by Walker *et al*.^16,17,31^. Although subjective analysis is an easy and quick method to analyse skeletal remains, it is prone to relatively large errors^32^. More objectively, colour can be measured by a technique called colourimetry, which is used in different research disciplines to objectively assess the colour of materials^33,34^. Colour can be measured in different colour systems. Research on HI changes of the colour of bone have focussed on changes in the Red, Green and Blue colour space (RGB), or on changes in the lightness (L*), red versus green (A*), and yellow versus blue (B*) colour space (L*A*B*). The majority of these studies used non-human bone and/or investigated a limited span of exposure duration which varied due to ramping and in-furnace cooling. The latter hampers the interpretation of experiments with short exposure durations. Moreover, most studies had a small number of samples per subgroup (see Table 1 for an overview) and therefore the variation within each subgroup, for both the RGB and L*A*B* system, is unknown. Furthermore, the effect of soft tissues, as heat transfer medium, is currently understudied^29^. As are the effects of additional variables, such as skeletal element, and sex and age of the deceased. So far, no objective colourimetric method to estimate the exposure temperature of thermally altered human skeletal elements has been published.

**Table 1:** Overview of literature on colourimetric analysis of thermally altered skeletal remains, including used material, methodology, sample size and key findings on HI changes in colour.

Authors	Material	Burning method	N total/N subgroup	Analysis method	Results	Ref.
Bonucci & Graziani (1975)	Archaeological human, archaeological non-human, and fresh non-human bone of 1 specie, modifications not provided.	Human cremated remains analysed from an archaeological context. Fresh non-human bone heated, unknown method, to 105 °C, 200 °C, 300 °C, 350 °C, 450 °C, 550 °C, 650 °C, 750 °C, 900 °C.	Not provided, at least N = 9/N = 1 for fresh non-human samples. (Subgroups based on temperature.)	Modifications not provided, subjective analysis; colour descriptions.	5 stages identified, provided colour descriptions. Associated temperatures based on the slopes of the thermogravimetric analysis.	^62^
Shipman *et al*. (1984)	Fresh non-human bone, unmodified, of 2 species.	Muffle furnace, 11 pre-set temperatures (unheated, 185 °C, 285 °C, 360 °C, 440 °C, 525 °C, 645 °C, 675 °C, 745 °C, 800 °C, 870 °C, 940 °C), sample was in the oven during ramping, exposure duration 240 min. (excl. ramping), cooled in furnace for 240 min.	N = 60/N = 5 (Subgroups based on temperature.)	Unmodified bone, surface assessed. Subjective analysis; colour descriptions and Munsell colour chart comparison.	5 stages identified, provided colour descriptions and associated Munsell colour codes. Associated temperatures based on ranges of the oven.	^32^
Nicholson (1993)	Archaeological bone and fresh non-human bone, unmodified, of different species (N = 6).	Muffle furnace, temperature range between 200 °C to 900 °C divided in steps of 100 °C with in addition an unheated group, exposure duration 150 min. No information provided on preheating or cooling down conditions.	N = 162/N = 3 (Subgroups based on temperature and species.)	Unmodified bone, surface assessed. Subjective analysis; Munsell soil colour chart.	Temperature associated dominant and minor colour codes provided. Differences found between species.	^57^
Walker *et al*. (2005/2008)	Fresh human bone. Femoral diaphysis, small sections with an approximate weight of 1.5 g.	Muffle furnace, temperature range between 100 °C to 1200 °C divided in steps of 100 °C, exposure durations of 60, 120 and 180 min. Samples heated in air and 2 types of soil as media. No information provided on preheating or cooling down conditions.	Not provided, at least N = 108/N = 1. (Subgroups based on temperature, duration and surrounding medium.)	Unmodified sample, surface measured. Colourimetric data collected in RGB by means of a flatbed scanner.	Variables temperature, duration and surrounding media (thus oxygen availability) all contribute to the changes in colour.	^16, 17^
Devlin *et al*. (2008)	Archaeological cremated human bone.	No samples experimentally exposed to heat, remains analysed from an archaeological context.	—	Unmodified sample, surface measured. Colourimetric data collected in L*A*B* by means of a flatbed scanner.	Variation in discolouration mapped for the archaeological site. Subjective interpretation of temperature.	^53^
Fredericks *et al*. (2015)	Fresh non-human bone, transverse sections, of 1 specie.	Muffle furnace, temperature range between 39 °C and 1000 °C divided in 23 subgroups, sample was in the oven during ramping, exposure duration 120 min. (excl. ramping), cooled at room temperature.	N = 69/N = 3 (Subgroups based on temperature.)	Bone milled to powder, powder measured. Colourimetric data collected in L*A*B* by means of a colourimeter.	The A* and B* coordinate changed in a similar fashion while the L* coordinate showed a different trend. Relative low standard deviations for the subgroups.	^15^
Wärmlander *et al*. (2019)	Archaeological human bone that was experimentally cremated & fresh non-human bone, unmodified.	Muffle furnace, temperature range between 400 °C and 1000 °C, divided in steps of 200 °C, sample was in the oven during ramping, exposure duration 60 min. (excl. ramping), cooled in furnace for 240 to 480 min.	N = 13/N = 5 archaeological bone, N = 4 fleshed non-human and N = 5 defleshed non-human.	Unmodified bone, surface measured. Colourimetric data collected in L*A*B* by means of a spectrophotometer.	The data from the fleshed and defleshed samples led to identifiable clusters, archaeological bone showed a different discolouration after cremated when compared to fresh bone.	^30^

*Table is not meant to be exhaustive but presents a selection of the available literature, extracted information is focussed on colourimetry while the majority of the studies combined colour analysis with additional investigative means.

Colourimetry is preferred over subjective analysis in order to meet the standards of Frye and Daubert for admissibility in court, as was advised by the National Research Council and acknowledged by the scientific community^35–38^. Variations in colour due to HI changes have to be known in order to be able to differentiate between different temperatures. We will study whether colourimetric analysis, based on RGB or L*A*B*, can be used to estimate the exposure temperature and test which colour system provides the most discriminating parameters. Additionally, we investigate the contribution of the additional variables (sex, age, skeletal element, surrounding medium, duration of exposure) to the observed HI changes of in bone colour parameters. To answer these research questions, a set of heat-exposed human bone samples was created. Because multiple variables had to be taken into account, categories within the set were based on exposure temperature, duration, the presence or absence of soft tissue, skeletal element, and sex and age of the deceased. Colourimetric data were collected and statistical analysis was performed to determine the effects of additional variables and to develop a decision model for estimating the exposure temperature. Finally, the precision was determined and the accuracy of the model was tested for both laboratory (by using a flatbed scanner) and field (by using a Digital Single Lens Reflex camera, DSLR) situations to determine whether the model meets the Frye and Daubert standards^35,36^.

## Materials and Methodology

### Sample collection and heating procedure

Upper extremities were collected from 10 fresh human cadavers that were donated to science; arms were used because of availability within the body donation program (see section *Ethical and legal framework* for details on the body donation program). All donations fell within the “old” adult age category (4 males aged 56, 66, 79, 87, and 6 females aged 54, 65, 69, 75, 81, 91)^39^. Cadaveric material from deceased with known bone pathology, or orthopaedic implants in the respective bones, was excluded from this study. Radii, ulnae, and humeri were extracted, manually defleshed, and stored in a refrigerator at a temperature between 4 to 7 °C. The long bones were sawn into sections with a bone saw immersed in a layer of water, to reduce heating due to friction^19^. The size of the samples ranged from approximately 4 mm (transverse diaphyseal slices, total 1088) and between approximately 40 mm to 80 mm (larger sections from the epiphyseal ends and diaphyseal sections, total 50). The majority of the samples were taken from the diaphyseal part and consists out of cortical bone, which is less fragile than the cancellous bone of epiphyseal ends. The samples were divided over subgroups, including; a series of temperature – exposure duration combinations, two different surrounding media, and two different section sizes. Samples were heated in porcelain cups in a preheated muffle oven (with an accuracy of ±2 °C). Exposure temperature ranged from room temperature to 900 °C with an exposure duration ranging between 5 to 50 min. Samples were exposed to heat in media plain air or wrapped in a layer of adipose tissue (*Sus scrofa domesticus*) of approximately 9 mm (sample was covered with adipose tissue on all sides) to mimic the presence of soft tissue. Heating in adipose tissue as medium was limited to 450 °C because at higher temperatures autoignition led to flaming combustion and hence unstandardized and uncontrollable heat exposure. The samples were left to cool down to room temperature outside of the muffle oven on a stone plate under atmospheric conditions. Personal protective equipment was worn and samples were handled with tweezers or crucible tongs to prevent contamination.

The set consisted of 1138 bone samples. This set was divided into a learning set and a test set. Skeletal remains of an individual were used for either the learning set or the test set, not for both. The learning set, which was used to develop a decision model to estimate exposure temperature, consisted of 833 transverse diaphyseal slices, with a minimum of N = 10 samples per temperature-duration subgroup. The test set was used to validate the model and consisted of 305 samples, with a minimum of N = 5 samples per subgroup. Power analysis showed that a deviation of 5% from the desired 100% correct categorisation could be determined with a sample size of about 150 samples (significance level 5%, power 0.8). Therefore, the test set consisted of 155 transverse diaphyseal slices, heated within the temperature-duration range of the learning set. The test set also included 50 samples categorized as larger sections (epiphyseal ends and diaphyseal sections), 50 transverse diaphyseal slices of approximately 4 mm that were heated for 5 min., and 50 transverse diaphyseal slices of approximately 4 mm that were heated for 50 min. ESM-1 (Tables S1 and S2) contains details on the sample collection and sample sizes per subgroup for both the learning and the test set.

### Data collection

All 1138 transverse slices (833 learning set and 305 test set) were scanned with a flatbed scanner at 300 dpi on a white background. Files were saved as uncompressed TIFF files. The learning set was scanned with an Epson scanner (Epson Stylus DX4200) and, as part of the validation process, the test set with a HP scanner (HP Photosmart C4680)^40^. The flatbed scanners were calibrated with an X-rite Munsell colourchecker Classic. ESM-2 (Tables S3 and S4) shows the difference between the true colour value and values obtained with the scanners, as well as the dynamic range of the scanners.

The 155 transverse slices heated between 10 to 30 min., 50 transverse slices heated for 50 min., and 50 larger sections (epiphyseal ends and diaphyseal sections) of the test set, already scanned with the flatbed scanner, were photographed on a white background with a Nikon D700 (DSLR) combined with a Nikon 35 mm Af-D F2.8 lens. Photos were taken in RAW format and white balancing was performed in Adobe Lightroom by means of the X-rite Munsell colourchecker Classic (ESM-2 Table S5 shows the dynamic range and difference between the true colour value and the values obtained with the DSLR). Subsequently, precision colour calibration was performed, and photos were saved as 300 dpi uncompressed TIFF files (see ESM-2).

Colourimetric data were collected from the image files with image processing software ImageJ. To prevent incorrect measurements due to overexposure and chromatic aberration, the transverse and periosteal surfaces were selected in the image excluding a rim of 1 to 2 mm from the outer skirts. The mean RGB and L*A*B* values were collected from the selected surface area. See ESM-2 for details on using ImageJ for collecting colourimetric data.

### Model development

Statistical analysis was performed in Microsoft® Excel for Mac 2016 and SPSS statistics for Mac. Measurement values of the learning set were used to test the correlation (Pearson) between the RGB and L*A*B* parameters. Parameters that correlated poorly with one another were plotted in a 2D scatterplot for cluster analysis. The correlation coefficient was interpreted according to Evans (1996)^41^. The clusters were manually identified and thresholds were set in-between clusters. This set of thresholds, derived from the data in the learning set, formed the decision model. The precision per cluster was determined from the minimum and maximum temperatures. The cluster associated temperature ranges were compared to the HI-stages and temperatures from literature (Correia (1997), Thompson (2005), Ellingham *et al*. and references therein (2014)) to check for discrepancies^21,22,29^. For subsequent statistical analysis age was grouped into 3 categories: below 60, from 60 to 80 and above 80. Multivariate analysis of variance (MANOVA) and multiple linear regression (MLR) were performed on the defined parameters based on the learning set, to test:

- Whether there is a significant effect of, or a significant interaction between, the different additional variables. (MANOVA)
- Whether exposure temperature, duration, surrounding media, skeletal element, and sex and age group of the deceased contributed significantly. (MLR)
- Which independent variables better explain the change of the parameter and if one of these independent variables had a greater effect than the others. (MLR)

MLR was performed on the complete learning set for exposure temperature and duration, and on a subset up to 450 °C for exposure temperature, duration and surrounding media. Statistical significance was accepted at p < 0.05.

### Validation of the model

The decision model was used to estimate the exposure temperature of the 305 samples of the test set, based on image files from the flatbed scanner (transverse slices, HP scanner) or the DSLR (transverse slices and epiphyseal ends). A different flatbed scanner, comparable in ease of use and output quality but different in year of production (Epson Stylus DX4200 produced in 2005 and HP Photosmart C4680 produced in 2009), was used in order to test the robustness of a calibrated colourimetric approach and the reliability of the decision model. A score of [1] was assigned when the temperature was correctly estimated, and in case of incorrect estimation a score of [0] was assigned. The percentage ‘correct temperature’ was calculated for the partitions of the test set and a mean percentage was calculated to reflect the overall accuracy of the proposed decision model.

## Results

### Development of decision model

**Figure 1:**
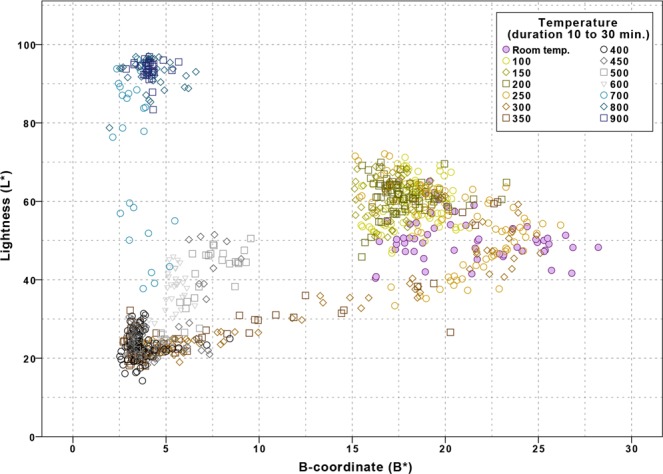
2D scatterplot based on L* and B* values of the 833 samples in the learning set, heated to temperatures ranging from room temperature to 900*C, for a duration of 10 to 30 min., in air or adipose tissue. (*This figure is best viewed online, in colour and at high resolution*.).

The RGB and L* values obtained from the learning set followed a similar temperature-dependent trend and showed a very strong and significant correlation (r between 0.918 and 0.988, p < 0.05), see ESM-3 Figs S3 and S4. This indicated that RGB and L* values provided similar discriminative power. Therefore, subsequent analysis was performed within the L*A*B* colour space. The correlation between L* and A* was the lowest (r = 0.136, p < 0.05), but only slightly less than the correlation between L* and B* (r = 0.297, p < 0.05) whereas the correlation between A* and B* was very strong (r = 0.896, p < 0.05). The 2D scatterplots showed that the data were most spread out when L* was plotted against B*, indicating that these two parameters provided the best information to identify clusters (see ESM-3 Tables S6 and Fig. S5). Therefore, the decision model was developed in L* versus B* data space (Fig. 1).

**Figure 2:**
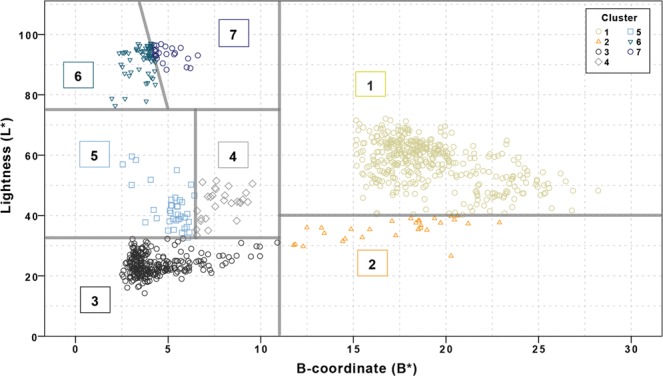
2D scatterplot of the L* and B* values of the 833 samples heated in both air and adipose tissue to temperatures ranging from unheated to 900 °C, for a duration of 10 to 30 min., grouped in seven clusters based on the rules of the proposed decision model (see Table 2). The gray lines correspond to thresholds defined in Table 2. Clusters are numbered according to the application of the decision rules.

**Figure 3:**
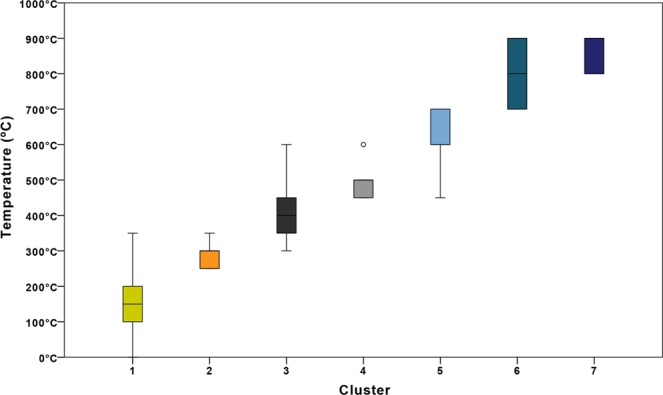
Boxplot of the temperature ranges for the 7 clusters that resulted from the application of the decision rules based on the observed L* and B*-coordinate values of the samples in the learning set (see Table 2, Figs 1 and 2).

In the 2D scatterplot of L* against B* seven mutually exclusive clusters could be identified. Clusters 1 to 5 were separated by a series of decision rules (Table 2) that separate the L* versus B* dataset with straight lines based on thresholds on either L* or B* values. Finally, cluster 6 and 7 were distinguished by a linear relation based on L* as well as B*. This decision model was then used to cluster the samples in the learning set and to plot their cluster membership (Fig. 2). The temperature ranges per cluster were determined from the included samples and shown to ascend in sequential order. The precision of clusters 1, 3, 5 and 6 is relatively low while the precision of clusters 2, 4, and 7 is relatively high (Fig. 3). No discrepancies were found when comparing the cluster associated temperature ranges with the HI-stages and temperatures from literature. See Table 2 for an overview of the clusters based on the decision model with associated minimum and maximum temperature and HI-stage.

**Table 2:** Proposed decision model.

Cluster	Decision rule^#^	Minimum temperature	Maximum temperature	HI-Stage
1	L > 40 and B > 11	0 °C	350 °C	Unheated - carbonization
2	L < 40 and B > 11	250 °C	350 °C	Carbonization
3	L < 32.5 and B < 11	300 °C	600 °C	Completely charred
4	[32.5 < L < 75] and B < 11	450 °C	600 °C	Inversion
5	[32.5 < L < 75] and B < 6.5	450 °C	700 °C	Inversion - calcination
6	L > 75 and B < 11 and L < (−25*B + 200)	700 °C	>900 °C^##^	Completely calcined
7	L > 75 and B < 11 and L > (−25*B + 200)	800 °C	>900 °C^##^	Completely calcined

The table gives the determined threshold values for 7 clusters including description. Further, the HI-stages associated with the clusters are shown. See Fig. 2 for a scatterplot of the cluster assignments based on L* and B* parameters and the boundaries between the respective clusters in the L* and B* parameter space. See Fig. 3 for a boxplot of the temperature distribution per cluster.

^#^For clarity, L* and B* are written as L and B.

^##^900 °C or higher, since 900 °C was the highest temperature used for this study.

There were no significant effects and interactions between the additional variables sex, age group, and skeletal element, on the parameters L* and B* for the continuous range of exposure temperatures above 300 °C (see Tables S7 and S8 in ESM-3 for the MANOVA results). Therefore, the effects of age, sex, and skeletal element can be ignored when the HI changes in colour are interpreted. MLR analysis showed that both the exposure temperature and duration individually contribute significantly to the outcome parameters L* and B* (Table 3). However, the partial correlation shows that changes in L* and B* are better explained by exposure temperature than by duration and the larger standardized beta coefficient shows that exposure temperature has a larger effect than duration on both L* and B* (Table 3). Up to a temperature of 450 °C exposure temperature, duration and media individually contribute significantly to the parameter L* but only exposure temperature contributes significantly to parameter B*. For both L* and B* parameters, exposure temperature better explains changes in L* and B*. Also, exposure temperature has a larger effect than duration and surrounding media (Table 4).

**Table 3:** MLR analysis on the complete learning set for parameters L* and B* based on exposure temperature and duration (N = 833).

Parameter	Independent	Std. Coefficients β	Correlation Part (semi-partial)	P
L*	Temperature	0.233	0.227	<0.000
	Duration	−0.079	−0.077	0.020
B*	Temperature	−0.717	−0.699	<0.000
	Duration	−0.090	−0.088	<0.000

**Table 4:** MLR analysis on subset of the learning set, based on exposure temperature up to 450 °C, duration and surrounding media (N = 671).

Parameter	Independent	Std. Coefficients Beta	Correlation Part (semi-partial)	P
L*	Temperature	−0.757	−0.716	<0.000
	Time	0.071	0.067	0.01
	Media	0.072	0.072	0.006
B*	Temperature	−0.761	−0.719	<0.000
	Time	−0.018	−0.027	0.483
	Media	0.032	0.032	0.198

### Validation of decision model

The proposed decision model was applied to the test set and the accuracy of the estimated exposure temperatures was determined, see Table 5. A mean accuracy of 94% was achieved for data acquired with the flatbed scanner (N = 305, Table 5). The accuracy was slightly lower but still acceptable when exposure duration deviated from the exposure duration of the samples in the learning set. Furthermore, a mean accuracy of 95% was achieved after the decision model was applied to data acquired with the DSLR (N = 255, Table 5). For colourimetric analysis the periosteal surface of the bone was measured because this surface is accessible without destruction of the bone fragment. However, in most of the larger diaphyseal sections it was observed that the colour of the periosteal surface differed from the internal colour. Samples heated around 400 °C exhibited carbonization and charring on the periosteal surface while the internal colour was lighter. Similarly, the periosteal surface already started to calcine when the internal structure was darker.

**Table 5:** Results of the application of the proposed decision model on the test set. Accuracy was determined for the different groups of samples imaged with the flatbed scanner or the DSLR camera.

Test set	Flatbed scanner		DSLR	
	N	Accuracy	N	Accuracy
Transverse slices approximately 4 mm, exposed to temperatures in the range of room temperature to 850 °C, with exposure duration between 10 to 30 min.	155	100%	155	97%
Transverse slices approximately 4 mm, heated in the range of 100 °C to 850 °C, exposed for 5 min.	50	90%	NA	NA
Transverse slices approximately 4 mm, heated in the range of 100 °C to 850 °C, exposed for 50 min.	50	90%	50	86%
Sections of approximately 40 mm to 80 mm, heated in the range of 100 °C to 850 °C, exposed for 30 min.	50	94%	50	96%
Total:	305	94%	255	95%

## Discussion

The current analyses show that a model of decision rules based on the L* and B* colourimetric values can be used to estimate the exposure temperature of bone fragments with an accuracy of more than 90%. In an experimental set of heat-exposed bone fragments high correlations were found between R, G, and B values whereas the L* value followed a very similar trend, as was expected since changes in the RGB channels are based on changes in L*. The variation of the colourimetric parameter values within the temperature subgroups was relatively small (see ESM-3 Figs S3 and S4), which shows that the chosen sample size was sufficient to determine precisely HI colourimetric changes. The trends in L*A*B* data observed in the current study correspond to the results obtained by Fredericks *et al*. (2015) despite the differences in exposure duration and their non-standard stepping of the temperature in the range between 39 °C and 1000 °C^15^. Comparison of the data of the current study with those of Fredericks *et al*. (2015) shows that HI changes occur within the first 30 minutes (min.) for the majority of the temperature groups^15^.

By means of the developed decision model, the exposure temperature range can be estimated, independently from exposure duration. The exposure temperature used for this study was limited to 900 °C although temperatures during fires can exceed 900 °C. It is to be expected that higher temperatures will give the same HI changes after a shorter duration of exposure than lower temperatures. Therefore, the maximum temperature of the final clusters, clusters 6 and 7, is probably not limited to 900 °C. The experimental design enabled the testing of the individual contribution of exposure temperature, duration and surrounding medium to the HI changes. This analysis showed that temperature contributes the strongest to the HI changes for parameters L* and B*. The contribution of exposure duration and surrounding medium is substantially lower whereas sex, age and skeletal element had no significant contribution. This substantiates the choice to fine-tune the decision model to estimate temperature and to accept the slight decrease in precision that comes from the exclusion of these additional variables from the model. Although the proposed decision model is based on transverse slices of approximately 4 mm thickness, application of the model to samples varying in size between 40 to 80 mm led to only a slight reduction in accuracy, which shows the robustness of the decision rules. Exposure of whole bones to heat results in fracturing of the bone; the size of the resulting fragments corresponds to the section sizes we used and tested.

For this study bone samples were exposed to heat in a muffle oven. In this way, the exposure to heat is almost uniform which differs from the profound temperature inhomogeneity during, for example, a house fire. Nonetheless, based on previous experiments it can be expected that similar HI changes will occur^10,19^. The next step would be to test the decision model on samples that have been exposed to heat by means of direct contact with flaming combustion. During flaming combustion oxygen levels fluctuate, leading to fluctuations in temperature. Therefore, it is expected to find more variation in HI colour changes, even in the same bone sample. When this variation is included in the decisions, by taking the minimum and maximum temperature of multiple clusters (as can be seen in ESM-2, Fig. S2), it is expected that the proposed model will result in the correct estimation of the temperature range that bone has been exposed to during such a fire incident. It is also possible to use the local cluster assignment to create a heat map, which then represents the thermal gradient that bones have been exposed to.

The human cadaveric material used in this study originated from both males and females in the age range of 54 to 91. Although material from individuals with known bone pathology was excluded, it cannot be ruled out, when considering the age range, that bones were included with a degree of osteopenia or osteoporosis. It is known that bone mineral density and collagen integrity decrease and bone porosity increases with increasing age^42–45^. Furthermore, lifestyle factors can also have an effect on bone integrity; for example, drugs metabolites can be stored within the cortical bone and the medullary cavity^46^. These individual differences can have an effect on the outcome in HI colour change. Changes of the bone microstructure lead to changes in the heat transfer rate. A decrease in collagen integrity might lead to a higher combustibility. An increase in porosity and decrease in bone mineral density might lead to an increase in oxygen availability which speeds up the process of carbonization and calcination. The fact that we saw no significant effects of age and sex shows that the first two issues are negligible when compared to the destructive influence of fire. The bone density can be expected to have a stronger influence because oxygen availability is a confounding factor for HI colour changes^13,16,17^. Adolescents, and younger, (<17 years old, with a degree of remodelling classified as histomorphological phase 3 or lower) have less advanced bone remodelling which might result in opposite effects^47^. The proposed model is, therefore, at this moment, only applicable to the remains of human adults.

In rare cases, decomposition is already an ongoing process prior to the fire. Little is known about the effect of heat or combustion on decomposing human remains, especially on the HI changes in colour of bone^48^. Based on the experiment of Wärmlander *et al*.^30^ it can be concluded that skeletal elements from an archaeological context, when exposed to heat, do not necessarily become charred^30^. This is related to the degree of preservation of the collagen within the bone matrix. Therefore, it is important to apply the proposed decision model only to situations in which the (pre-)burning conditions are known or can be deduced from additional information. Discolouration due to staining from organic matter, bacterial or fungal activity or the presence of metals needs to be taken into consideration when dealing with remains that have not been collected within a limited time frame after the fire incident^49–51^. In such cases substantiation of the thermal discolouration is needed. This applies to forensic as well as archaeological contexts.

As yet it is unknown whether differences in mineral density, collagen integrity, porosity, the presence of drug metabolites, the state of preservation of the remains, or a combination of the previous, significantly contribute to the outcome in HI colour change. However, when considering the temperature ranges associated with the temperature clusters, it is expected that the effect of these individual differences will not extend beyond the boundaries of each temperature cluster.

Although the destruction of human remains (under optimal fire conditions) follows a predictable sequence, remains from fire incidents can be difficult to interpret^8,12^. This is caused by the unknown peri-mortem conditions of the deceased, i.e. (but not limited to) preservation, postmortem interval, and degree of dismemberment^52^. L* and B* values obtained from the periosteal surface, or outer surface in case of fragmentation, in most cases will represent the maximum temperature that bone has been exposed to. However, surface characteristics do not necessarily represent the degree of HI changes of the internal structure^53^. This is especially important when assessing the feasibility of obtaining DNA- or isotope profiles from recovered bone fragments. Although it is thought that such sampling will be fruitless in case of charring^54^, it has recently been established that it is possible to extract DNA of sufficient quantity and quality from thermally altered bone that has been heated to a temperature of 200 °C, corresponding to cluster 1 in our model^14,15^. Schwark *et al*. (2011) even obtained DNA profiles from carbonized bone, although highly degraded, and sporadically from bone in the inversion stage (associated with blue-grey), which corresponds to clusters 2 to 4, respectively^55^. Evidently, the methodology for DNA extraction and amplification has not yet been optimized for burned skeletal remains^56^. Finally, it is important to realize that the outer surface of a bone can be contaminated with soot deposits, thus mimicking charred bone and possibly leading to a biased interpretation of HI changes and an erroneous exposure temperature. This also holds for temperature estimation by means of the model proposed in this paper.

In the application of the proposed decision rules it is essential to standardize lightning conditions and to colour calibrate precisely the image acquisition device. It is highly recommended to create a validation set of bone fragments to check calibrations settings. Due to different legal frameworks and ethical considerations it may not be possible in every country to create such a reference set of human remains. So far, it is not yet certain whether animal bone can serve as a proxy for human bone, and if so, which animal should be used, especially since Nicholson (1993) found differences between species^57^. Although, in general, mammalian bone is composed of the same chemical components, there are species differences in internal (micro-)structure as well as in the ratio between the organic and inorganic components. Whether these differences are discernible by means of colourimetry, or are negligible, is unknown.

Colourimetry is a secondary method. It is generally accepted that a primary method, like chemical component analysis, will result in a higher precision and accuracy^22^. Nonetheless, the results presented in this paper show that colourimetry is robust and the validation of the model shows that reproducible results can be obtained by using two different flatbed scanners and a DSLR. With this, the proposed decision model more closely meets the Daubert and Frye standards for forensic analysis^35,36^. Unlike most chemical component analysis techniques, colourimetry is a non-destructive technique, which is an important consideration from both a practical and ethical point of view for forensic as well as archaeological work. Combining colourimetry with other methods might lead to more detailed clusters and thus improved precision. In the current study the cluster analysis was done manually. Machine learning, as shown by Wärmlander *et al*.^30^, might further improve temperature estimations^30^. Other techniques can be combined with colourimetry to further substantiate the estimation of the exposure temperature. Among these techniques are the degree of luminescence, histological analysis and analysis of chemical composition by means of Fourier-transform Infrared Spectroscopy, Raman or spectral imaging^19,29,58–61^.

## Conclusion

Based on the results it can be concluded that the L*A*B* colour space provides more information about heat-induced colour changes of human bone samples than the RGB system. The parameters L* and B* were used to develop a model of decision rule that can be used to accurately differentiate between seven temperature clusters. The precision, expressed as the included temperature range, differs per cluster. The latter is caused by variables like exposure duration and surrounding medium. The accuracy of the developed method was high, even for samples that had a composition or size that differed from samples in the learning set.

We therefore conclude that colourimetry can be used to estimate the temperature that human skeletal remains were exposed to, especially when the context is known and when it is certain that the true colour of the bone, and not that of the pyrolytic by-products, is measured.

### Ethical and legal framework

The material used in the experiments was obtained through the body donation program of the Department of Medical Biology of Amsterdam University Medical Centres, location AMC, The Netherlands, in accordance with Dutch legislation (art. 67 Burial Act) and the guidelines of the medical ethical committee of the Amsterdam University Medical Centres, location AMC.

## Supplementary information

Electronic Supplementary Material

## Data Availability

All relevant data are available in this contribution, see ESM-4 Tables S9 and S10.
