# Supplementary material for: Colourimetric analysis of thermally altered human bone samples
Source: Sci Rep. 2019 Jun 20;9:8923. doi: 10.1038/s41598-019-45420-8 (PMC6586926; doi:10.1038/s41598-019-45420-8)
Supplement: Supplementary file 1 — Electronic Supplementary Material [file 41598_2019_45420_MOESM1_ESM.pdf]

Electronic Supplement: Colourimetric analysis of thermally altered human bone samples.

Tristan Krap [1,2,3,4], Jan M. Ruijter [2], Kevin Nota [4], Joyce Karel [4], A. Lieke Burgers [5], Maurice C.G. Aalders [5,6], Roelof-Jan Oostra [2], Wilma Duijst [1,3]

1. Maastricht University, Maastricht, The Netherlands
2. Amsterdam UMC, Location AMC, department of Medical Biology, section Anatomy, Amsterdam, The Netherlands
3. Ars Cogniscendi Foundation for Legal and Forensic Medicine, Wezep, The Netherlands
4. Department of Life Sciences and Technology–Biotechnology–Forensic Science, Van Hall Larenstein, University of Applied Sciences, Leeuwarden, The Netherlands
5. Amsterdam UMC, Location AMC, department of Biomedical Engineering and Physics, Amsterdam, The Netherlands
6. Co van Ledden Hulsebosch Center, Amsterdam, The Netherlands

Corresponding author: Tristan Krap
Mail: t.krap@amc.nl
Address: Amsterdam UMC, Location AMC,
department of Medical Biology, section Anatomy
Meibergdreef 15, 1105 AZ Amsterdam, The Netherlands

ESM-1 Sample population

Bone samples in the learning set were divided over 58 subgroups, including exposure temperature, surrounding media, and exposure duration, with a minimum of 10 samples per subgroup (table s1). Samples in the test set were divided over 61 subgroups, including exposure temperature, size of the sample, surrounding medium and exposure duration, with a minimum of 5 samples per subgroup (table s2).

Table s1. Heating scheme for the learning set, transverse slices heated in two different media.

Temperature:	Surrounding medium:	Duration (min.):	N (samples):
Unheated	-	-	58
100°C	Air<br>Adipose tissue	10/20/30<br>10/20/30	12/12/16<br>12/12/16
150°C	Air<br>Adipose tissue	10/20/30<br>10/20/30	12/12/12<br>12/12/12
200°C	Air<br>Adipose tissue	10/20/30<br>10/20/30	12/12/16<br>12/12/16
250°C	Air<br>Adipose tissue	10/20/30<br>10/20/30	10/12/16<br>11/12/16
300°C	Air<br>Adipose tissue	10/20/30<br>10/20/30	12/12/16<br>12/12/16
400°C	Air<br>Adipose tissue	10/20/30<br>10/20/30	12/16/12<br>12/16/12
450°C	Air<br>Adipose tissue	10/20/30<br>10/20/30	10/12/10<br>12/10/10
500°C	Air	10/20/30	10/10/10
600°C	Air	10/20/30	10/10/10
700°C	Air	10/20/30	10/10/10
800°C	Air	10/20/30	10/10/20
900°C	Air	10/20/30	10/10/10
Total:			833

Table s2. Heating scheme for the test set, heated transverse larger sections (diaphyseal parts and epiphyseal ends) and transverse slices that were heated for a longer duration, including the surrounding media.

Temperature:	Type:	Surrounding medium:	Duration (min.):	N (samples):
Unheated	Transverse section	-	-	5
100°C	Transverse section<br><br>Larger section	Air<br>Adipose tissue<br>Air	5/10/20/30/50<br>20/30<br>30	5/5/5/5/5<br>5/5<br>5
250°C	Transverse section<br><br>Larger section	Air<br>Adipose tissue<br>Air	5/10/20/30/50<br>20/30<br>30	5/5/5/5/5<br>5/5<br>5
300°C	Transverse section<br><br>Larger section	Air<br>Adipose tissue<br>Air	5/10/20/30/50<br>20/30<br>30	5/5/5/5/5<br>5/5<br>5
350°C	Transverse section<br>Larger section	Air<br>Air	5/50<br>30	5/5<br>5
450°C	Transverse section<br>Larger section	Air<br>Air	5/10/20/30/50<br>30	5/5/5/5/5<br>5
500°C	Transverse section<br>Larger section	Air<br>Air	5/10/20/30/50<br>30	5/5/5/5/5<br>5
600°C	Transverse section<br>Larger section	Air<br>Air	5/50<br>30	5/5<br>5
650°C	Transverse section<br>Larger section	Air<br>Air	10/20/30/50<br>30	5/5/5/5<br>5
700°C	Transverse section<br>Larger section	Air<br>Air	5/10/20/30/50<br>30	5/5/5/5/5<br>5
800°C	Transverse section	Air	5	5
850°C	Transverse section<br>Larger section	Air<br>Air	10/20/30/50<br>30	5/5/5/5<br>5
900°C	Transverse section	Air	5	5
Total:				305

<u>ESM-2 Data acquisition by means of ImageJ and colour calibration.</u>

The X-rite Munsell colourchecker Classic chart as well as all samples were scanned by means of a flatbed scanner (Epson for the learning set and HP for the data set, file type TIFF) and photographically recorded with a DLSR (Nikon D700, stored in file type RAW and converted to TIFF in Adobe Lightroom). The image files were loaded into ImageJ software as shown in fig. s1A (version 1.51j8; Java 1.8.0_112), and colourimetric data was collected with ImageJ procedures (toolkit: ijp-toolkit_bin.2.1.0).

In order to collect data in L*A*B* colours the image has to be converted with the following image processing step: in 'Plugins' select 'Color' and choose 'RGB to L*A*B* stack. The converted image is displayed in grayscale (fig. s1B). However, the data presented by the toolkit represents colourimetric data in L*A*B*, this can be verified by looking up the specific colour for the collected L*A*B* data. By means of the Selection Brush Tool the surface area of the sample can be selected. This selection excludes a few millimetres from the outskirts of the sample to avoid incorrect measurements due to overexposure and chromatic aberration at the edges of the image bone fragment. The L*A*B* data can be collected by selecting 'Measurement Bands', which can be found in the submenu 'Color' under 'Plugins'. The mean value of the L*A*B* parameters for the pixels in the selected surface was used for further analysis. In order to obtain RGB values the same steps should be taken without the conversion to L*A*B*. In case of colour differences due to differences in heat exposure it is possible to select the areas of the bone to determine the highest and lowest exposure temperature, as can be seen in fig. s2.

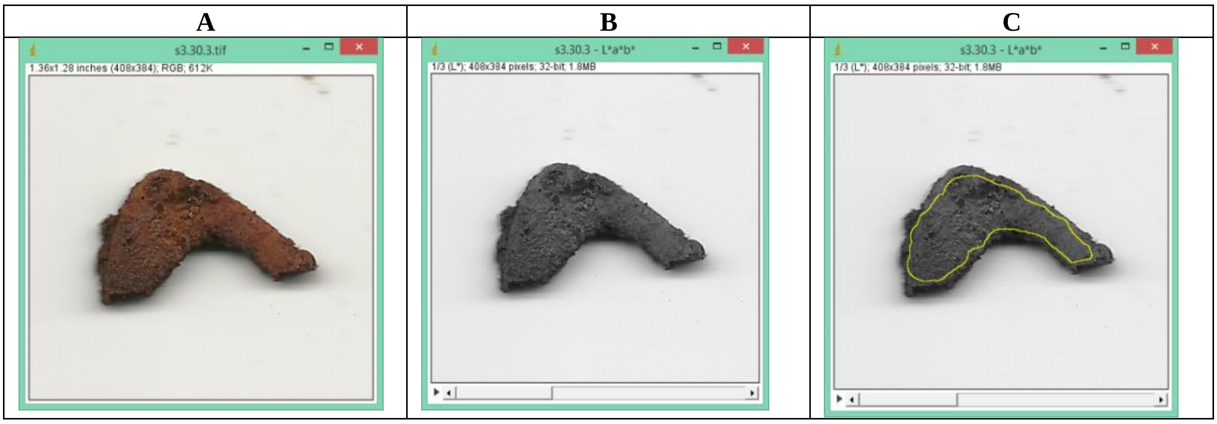

Fig. s1 Colour extraction in ImageJ based on a scan of a transverse cross section. A: the original image, B: the image converted to L*A*B*, and C: the selection of the area from which colourimetric data is collected.

A	B

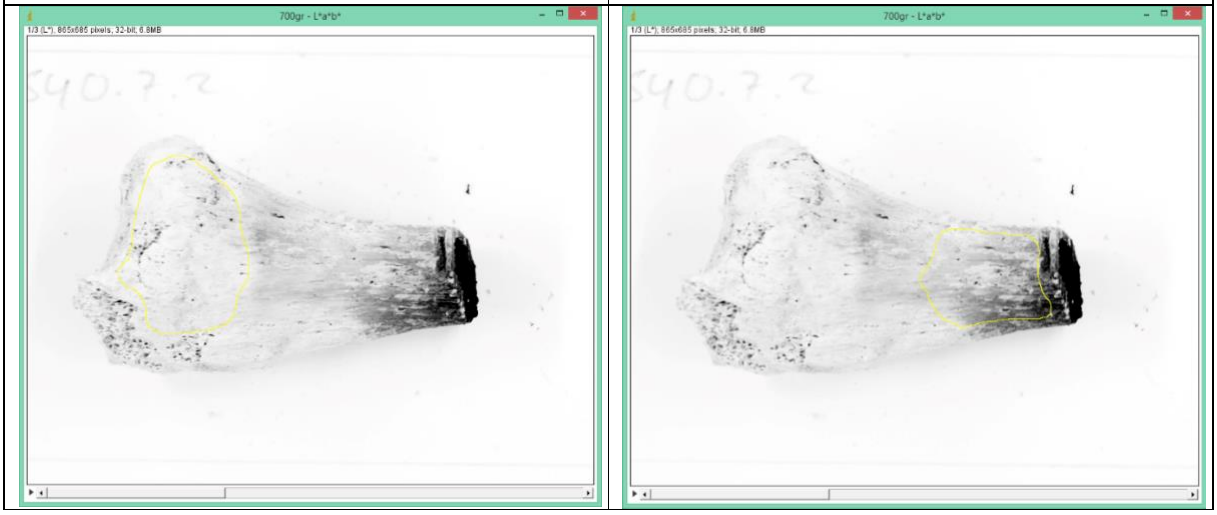

Fig. s2 Colour extraction in ImageJ based on a photograph of an epiphyses with a small part of the diaphysis. A shows the selection of the white part and B shows the selection of the grey part.

The devices (Flatbed scanners Epson and HP, and DSLR Nikon D700) were colour calibrated by means of an X-rite Munsell colourchecker Classic chart. The chart was scanned prior to scanning the samples, halfway through the scanning session and at the end of the scanning session. The dynamic range and specific colour values and coordinates were measured in Red (R), Green (G), Blue (B), Lightness (L*), A and B-coordinates (A*, B*) for the black, white and tile #2 of the chart. The mean of the values obtained in the three scans of the chart was calculated (see tables s3, s4 and s5), and subsequently measurements values of the bone samples were corrected by adding or subtracting the calculated mean.

Table s3. The dynamic range and colour of the flatbed scanner used for the learning set (Epson), based on black, white and tile #2.

	R	G	B	L*	A*	B*
Black						
Colourchart:	52	52	52	20,461	-0,079	-0,973
Scanner:	47,612	48,349	49,493	20,004	-0,024	-0,835
White						
Colourchart:	243	243	242	96,539	-0,425	1,186
Scanner:	239,501	240,111	239,906	94,710	-0,218	0,028
Tile #2						
Colourchart:	194	150	130	65,711	18.13	17,81
Scanner:	189,429	138,709	126,068	62,306	17,678	13,885

Table s4. The dynamic range and colour of the flatbed scanner used for the test set, based on black, white, and tile #2.

	L*	B*
Black		
Colourchart:	20,461	-0,973
Scanner:	20,985	2,256
White		
Colourchart:	96,539	1,186
Scanner:	99,650	3,772
Tile #2		
Colourchart:	65,711	17,81
Scanner:	65,8344	21,7182

Table s5. The dynamic range and colour of the Nikon D700 used for the test set, based on black white, and tile #2.

	L*	B*
Black		
Colourchart:	20,461	-0,973
Camera:	5,62	-3,32
White		
Colourchart:	96,539	1,186
Camera:	98,54	-0,13
Tile #2		
Colourchart:	65,711	17,81
Camera:	81,208	15,944

ESM-3 Results of learning set

Fig. s3 shows the colourimetric data for channels Red (R), Green (G), and Blue (B), based on 833 samples heated in the range of 100°C to 900°C for a duration of 10 to 30 minutes in two different media. Fig. s4 shows the colourimetric data for Lightness (L*) and the A- and B-coordinates (A*, B*), based on 833 samples heated in the range of 100°C to 900°C for a duration of 10 to 30 minutes in two different media.

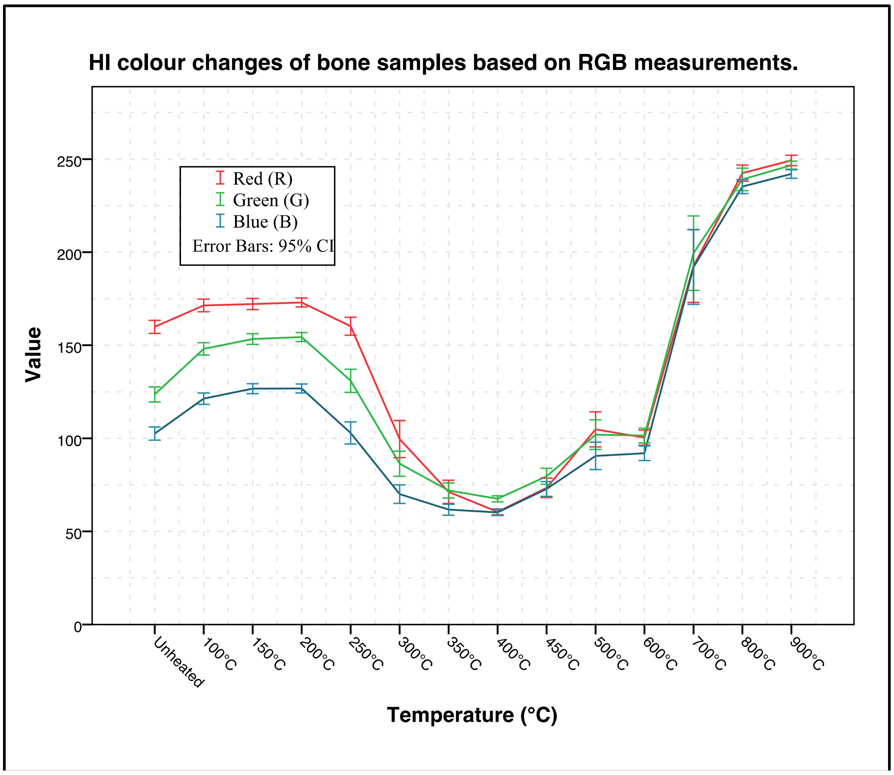

Fig. s3 HI colour changes of bone samples heated in the range of 100 - 900°C for 10 to 30 minutes in media air and adipose tissue measured in RGB (N=833, see ESM-1, table s1 for information on subgroups).

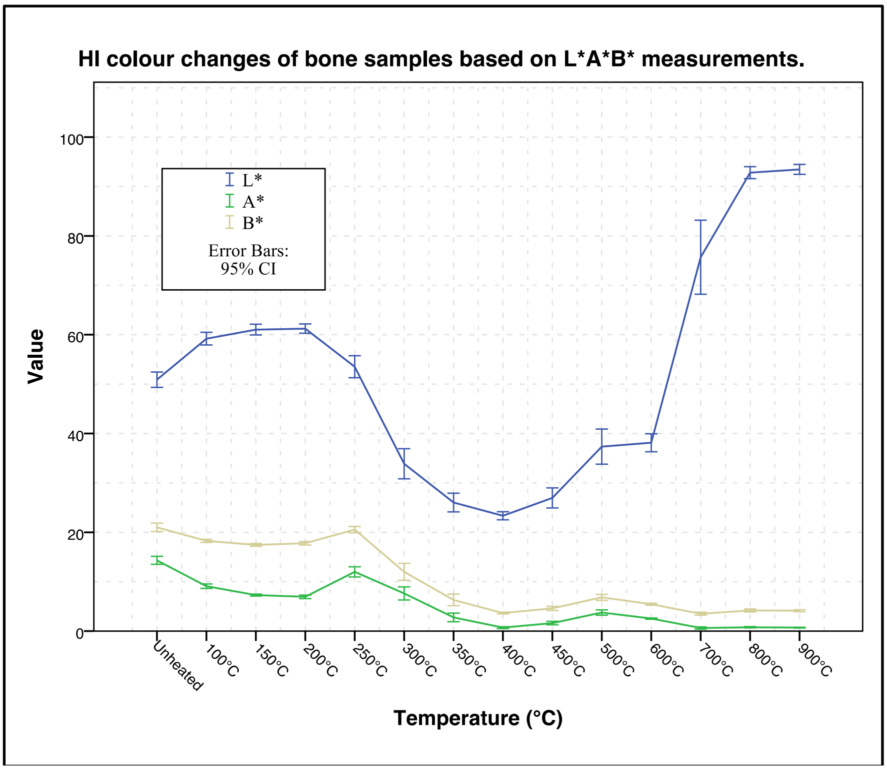

Fig. s4 HI colour changes of bone samples heated in the range of 100 - 900°C for 10 to 30 minutes in media air and adipose tissue measured in L*A*B* (N=833, see ESM-1, table s1 for information on subgroups).

There was a strong correlation between R, G, B and L*, based on the Pearson correlations. The correlation between R,G,B,L* on one hand and A* and B* on the other is considerately lower (table s6, also compare fig. s3 and s4). Therefore, the information in the RGB colour model and the L* channel of the L*A*B* colour model is very similar it was decided to continue with just the L*A*B* colour model. To illustrate the relations between L*, A* and B*, the measured data was plotted in 2D scatterplots. The data of L* plotted against B* was more spread out than L* plotted against A*, leading to more identifiable clusters (fig. s5).

Table s6. Pearson correlations between RGB and L*A*B* (learning set, N=833).

		**R**	**G**	**B**	**L***	**A***	**B***
**R**	Pearson Correlation	1	0,963	0,910	0,982	0,289	0,432
	Sig. (2-tailed)		0,000	0,000	0,000	0,000	0,000
	N	833	833	833	833	833	833
**G**	Pearson Correlation	0,963	1	0,981	0,988	0,038	0,201
	Sig. (2-tailed)	0,000		0,000	0,000	0,267	0,000
	N	833	833	833	833	833	833
**B**	Pearson Correlation	0,910	0,981	1	0,957	-0,100	0,029
	Sig. (2-tailed)	0,000	0,000		0,000	0,004	0,409
	N	833	833	833	833	833	833
**L***	Pearson Correlation	0,982	0,988	0,957	1	0,136	0,297
	Sig. (2-tailed)	0,000	0,000	0,000		0,000	0,000
	N	833	833	833	833	833	833
**A***	Pearson Correlation	0,289	0,038	-0,100	0,136	1	0,896
	Sig. (2-tailed)	0,000	0,267	0,004	0,000		0,000
	N	833	833	833	833	833	833
**B***	Pearson Correlation	0,432	0,201	0,029	0,297	0,896	1
	Sig. (2-tailed)	0,000	0,000	0,409	0,000	0,000	
	N	833	833	833	833	833	833

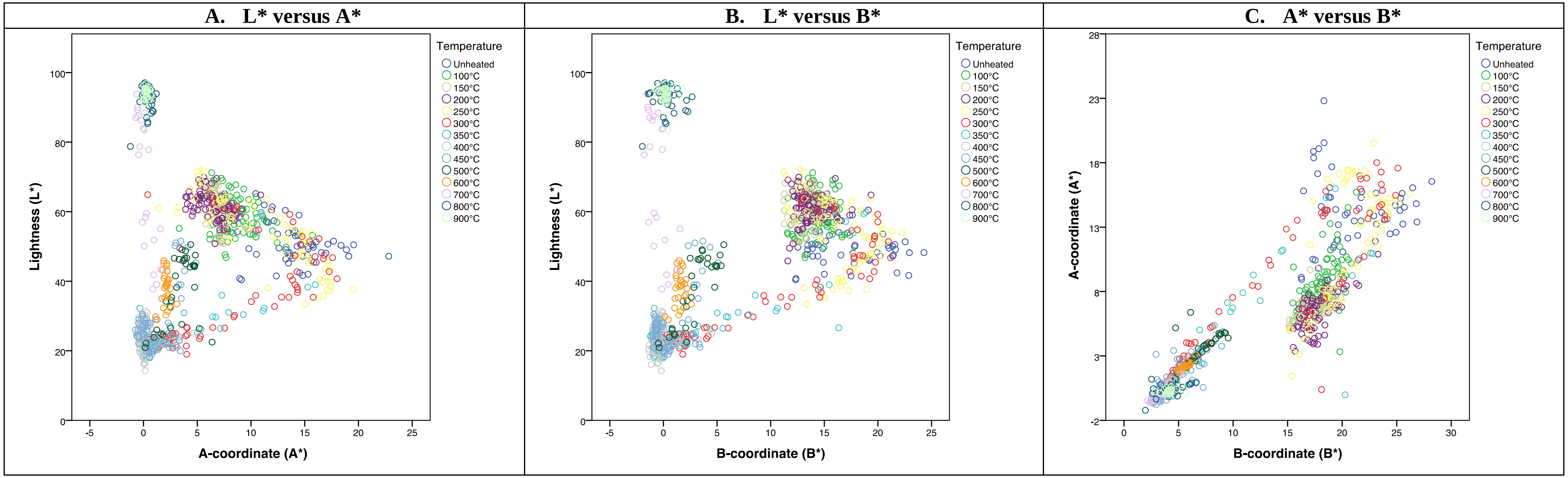

Fig. s5 2D scatterplots of the three parameters within the L*A*B* colour space. The data is most spread out when L* is shown against B* (B), slightly less when L is shown against A* (A), and least spread out when B* is shown against A* (C). See ESM-3 table 6 for associated correlations.

Testing the effects of the different additional variables in the data set.

To determine the effect of additional variables in the dataset on the variation of the colourimetric parameters a multivariate analysis of variance (MANOVA) was performed on the parameters L* and B* with the factors sex, age group, skeletal element, surrounding medium, duration and temperature. Missing categories in a multivariate design are known to bias the results of the MANOVA due to interpolation (fig. s6 and s7), Therefore the analysis was restricted to the samples with an exposure temperature above 300°C. The results of this MANOVA test are shown in table s7 (number of observations per factor) and table s8. Table s8 shows a significant effect of exposure temperature on L* and B* and a small effect of surrounding medium on B*. The effects of the other additional variables not approaching significance. The tests of tow and more-way interactions between the factors only show significance for some interactions involving exposure temperature. This test results shows that the effects of sex, age and skeletal element can be ignored in the analysis of the effect of exposure temperature on the L* and B* parameters in the colourimetric dataset. Because of the small effect of surrounding medium and its highly significant interaction with exposure temperature (both on the B* parameter) this factor was included in the multiple regression analysis (see main text).

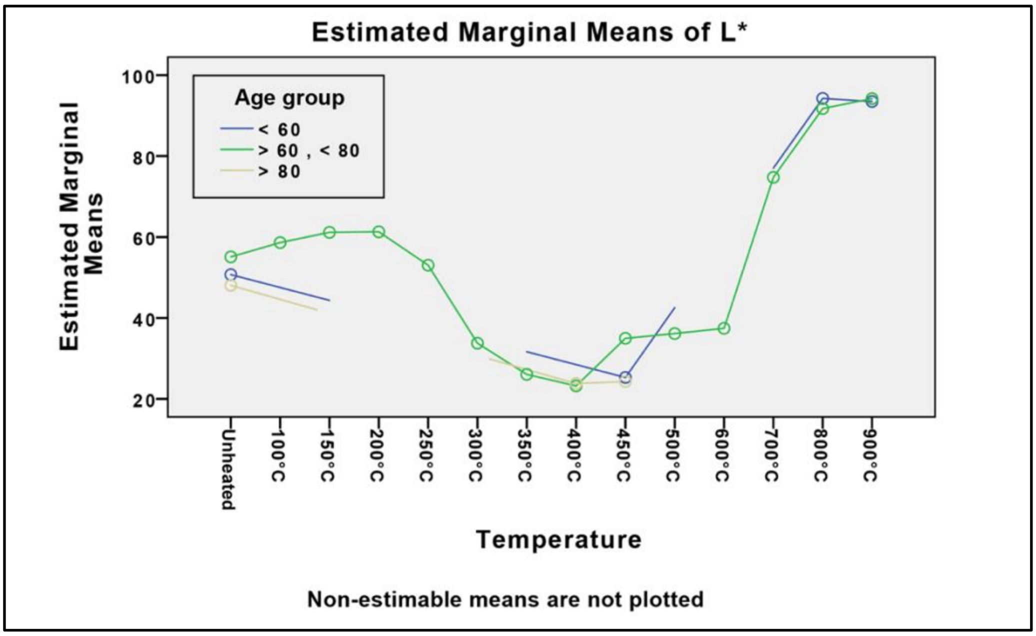

Fig. s6 Graph of the temperature dependent changes of the estimated marginal means of L*.

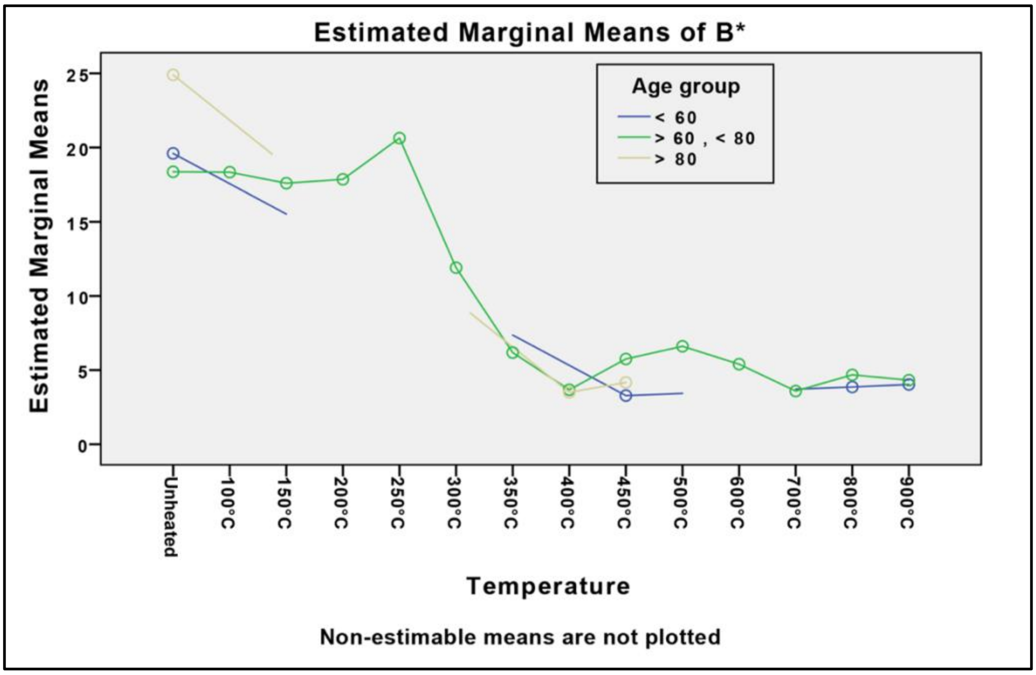

Fig. s7 Graph of the estimated marginal means of B* plotted against temperature.

Table s7. Number of observations per factor in the MANOVA on the learning set. Only samples with an exposure temperature above 300°C were included.

		Value Label	N
Sex	1	Male	274
	2	Female	112
Skeletal element	1	Ulna	151
	2	Radius	179
	3	Humerus	56
Media	1	Air	274
	2	Fat	112
Temperature	350°C		80
	400°C		80
	450°C		64
	500°C		32
	600°C		30
	700°C		30
	800°C		40
	900°C		30
Age group	1	< 60	46
	2	> 60 , < 80	276
	3	> 80	64

Table s8. Results of the MANOVA on the learning set. Only samples with an exposure temperature above 300°C were included.

Source	Dependent Variable	Type III Sum of Squares	df	Mean Square	F	Sig.
Sex	L*	86,006	1	86,006	1,369	,243
	B*	2,078	1	2,078	,325	,569
Skeletal element	L*	92,895	2	46,448	,739	,478
	B*	5,039	2	2,519	,394	,674
Media	L*	,238	1	,238	,004	,951
	B*	22,154	1	22,154	3,469	,063
Temperature	L*	118285,466	7	16897,924	268,892	,000
	B*	378,553	7	54,079	8,467	,000
Age group	L*	7,125	2	3,562	,057	,945
	B*	,426	2	,213	,033	,967
Sex * Skeletal element	L*	71,701	1	71,701	1,141	,286
	B*	2,832	1	2,832	,443	,506
Sex * Media	L*	37,366	1	37,366	,595	,441
	B*	,232	1	,232	,036	,849
Sex * Temperature	L*	1361,871	7	194,553	3,096	,004
	B*	59,566	7	8,509	1,332	,234
Sex * Age group	L*	,000	0	.	.	.
	B*	,000	0	.	.	.
Skeletal element * Media	L*	171,306	2	85,653	1,363	,257
	B*	1,287	2	,644	,101	,904
Skeletal element * Tmperature	L*	493,379	12	41,115	,654	,795
	B*	21,342	12	1,779	,278	,992
Skeletal element * Age group	L*	3,749	1	3,749	,060	,807
	B*	1,264	1	1,264	,198	,657
Media * Temperature	L*	189,071	2	94,535	1,504	,224
	B*	97,681	2	48,840	7,647	,001
Media * Age group	L*	13,756	1	13,756	,219	,640
	B*	,065	1	,065	,010	,920
Temperature * Age group	L*	69,760	2	34,880	,555	,575
	B*	3,095	2	1,547	,242	,785
Sex * Skeletal element * Media	L*	41,472	1	41,472	,660	,417
	B*	11,993	1	11,993	1,878	,172
Sex * Skeletal element * Temperature	L*	21,226	1	21,226	,338	,562
	B*	,475	1	,475	,074	,785
Sex * Skeletal element * Age group	L*	,000	0	.	.	.
	B*	,000	0	.	.	.
Sex * Media * Temperature	L*	36,569	2	18,284	,291	,748
	B*	18,255	2	9,128	1,429	,241
Sex * Media * Age group	L*	,000	0	.	.	.
	B*	,000	0	.	.	.
Sex * Temperature * Age group	L*	,000	0	.	.	.
	B*	,000	0	.	.	.
Skeletal element * Media * Temperature	L*	23,903	2	11,951	,190	,827
	B*	,167	2	,084	,013	,987
Skeletal element * Media * Age group	L*	4,637	1	4,637	,074	,786
	B*	,799	1	,799	,125	,724
Skeletal element * Temperature * Age group	L*	,000	0	.	.	.
	B*	,000	0	.	.	.
Media * Temperature * Age Group	L*	218,099	1	218,099	3,471	,063
	B*	5,489	1	5,489	,859	,355
Sex * Skeletal element * Media * Temperature	L*	26,832	1	26,832	,427	,514
	B*	2,424	1	2,424	,380	,538
Sex * Skeletal element * Media * Age group	L*	,000	0	.	.	.
	B*	,000	0	.	.	.
Sex * Skeletal element * Temperature * Age group	L*	,000	0	.	.	.
	B*	,000	0	.	.	.
Sex * Media * Temperature * Age group	L*	,000	0	.	.	.
	B*	,000	0	.	.	.
Skeletal element * Media * Temperature * Age group	L*	,000	0	.	.	.
	B*	,000	0	.	.	.
Sex * Skeletal element * Media * Temperature * Age group	L*	,000	0	.	.	.
	B*	,000	0	.	.	.
Error	L*	20675,242	329	62,843		
	B*	2101,250	329	6,387		
Total	L*	1038215,163	386			
	B*	12159,714	386			

R Squared = ,890 (Adjusted R Squared = ,872) b. R Squared = ,840 (Adjusted R Squared = ,815)

ESM-4 Colourimetric (calibrated) data learning and test set

Table s9. Raw data of the learning set (1/20)

ID nr	Sex	Age	Skeletal element	Medium	Temp. (°C)	Time (min.)	R	G	B	L*	A*	B*
1	Male	66	Humerus	Air	450	30	75,61	76,36	66,57	28,02	2,29	5,69
2	Male	66	Humerus	Air	450	30	94,70	91,42	80,44	35,04	3,51	7,16
3	Male	66	Humerus	Air	500	10	71,92	72,33	62,41	26,57	2,52	5,99
4	Male	66	Humerus	Air	500	10	105,89	98,97	86,07	38,26	4,75	8,70
5	Male	66	Humerus	Air	500	20	120,74	113,23	99,82	44,15	4,73	8,90
6	Male	66	Humerus	Air	500	20	120,89	113,78	100,52	44,57	4,60	8,73
7	Male	66	Humerus	Air	500	30	119,97	114,79	102,35	45,01	3,94	8,02
8	Male	66	Humerus	Air	500	30	124,84	119,31	106,71	46,67	4,03	8,06
9	Male	66	Humerus	Air	500	30	118,33	112,03	99,21	44,08	4,34	8,42
10	Male	66	Humerus	Air	500	30	120,69	113,34	100,01	44,46	4,70	8,87
11	Male	66	Humerus	Air	600	10	93,75	94,16	84,18	35,68	2,29	5,89
12	Male	66	Humerus	Air	600	10	95,16	95,61	85,46	36,16	2,27	6,00
13	Male	66	Humerus	Air	600	20	98,72	98,56	88,35	37,91	2,48	6,12
14	Male	66	Humerus	Air	600	20	101,58	102,24	92,16	39,31	2,20	5,90
15	Male	66	Humerus	Air	600	30	100,94	102,22	93,04	38,82	2,06	5,26
16	Male	66	Humerus	Air	600	30	111,14	111,79	102,31	42,94	2,21	5,51
17	Male	66	Humerus	Air	700	10	139,71	148,21	142,14	56,94	-0,11	2,55
18	Male	66	Humerus	Air	700	10	147,22	154,32	147,61	59,56	0,28	3,05
19	Male	66	Humerus	Air	700	20	224,74	234,63	228,68	89,25	-0,55	2,53
20	Male	66	Humerus	Air	700	20	228,42	238,26	232,33	90,03	-0,53	2,45
21	Male	66	Humerus	Air	700	30	244,47	250,81	242,62	94,54	0,19	3,98
22	Male	66	Humerus	Air	700	30	246,26	252,45	244,41	95,29	0,25	3,92
23	Male	66	Humerus	Air	800	10	233,75	142,44	235,76	91,59	-0,35	2,94
24	Male	66	Humerus	Air	800	10	237,74	243,31	234,41	92,11	0,32	4,39
25	Male	66	Humerus	Air	800	20	249,01	255,11	246,71	96,10	0,22	4,06
26	Male	66	Humerus	Air	800	20	249,90	255,91	247,34	96,40	0,21	4,08
27	Male	66	Humerus	Air	900	10	246,65	252,29	243,78	95,47	0,35	4,15
28	Male	66	Humerus	Air	900	10	247,61	253,29	245,06	95,34	0,37	4,05
29	Male	66	Humerus	Fat	0	0	154,49	118,27	95,87	49,39	15,33	18,79
30	Male	66	Humerus	Fat	0	0	156,45	122,04	100,25	50,65	14,60	18,08
31	Male	66	Humerus	Air	450	30	56,26	60,73	52,23	19,86	0,97	4,42
32	Male	66	Humerus	Air	450	30	59,86	64,62	56,37	22,51	0,98	4,33
33	Male	66	Humerus	Air	500	10	102,45	100,25	89,25	38,21	3,09	6,77
34	Male	66	Humerus	Air	500	10	107,31	106,31	95,97	41,53	2,97	6,56
35	Male	66	Humerus	Air	500	20	68,79	72,39	63,54	26,00	1,23	4,73
36	Male	66	Humerus	Air	500	20	94,62	93,82	83,02	35,36	2,62	6,53
37	Male	66	Humerus	Air	500	30	129,90	125,75	113,79	49,07	3,57	7,53
38	Male	66	Humerus	Air	500	30	129,91	125,19	113,39	48,74	3,92	7,57
39	Male	66	Humerus	Air	600	10	103,56	104,04	94,23	39,70	2,23	5,70
40	Male	66	Humerus	Air	600	10	105,49	106,01	96,37	40,30	2,25	5,52
41	Male	66	Humerus	Air	600	20	110,73	111,54	102,08	43,08	2,21	5,50
42	Male	66	Humerus	Air	600	20	116,24	116,90	107,19	45,09	2,17	5,63

Table s9. Raw data of the learning set (2/20)

ID nr	Sex	Age	Skeletal element	Medium	Temp. (°C)	Time (min.)	R	G	B	L*	A*	B*
43	Male	66	Humerus	Air	600	30	112,47	114,24	105,12	45,24	1,89	5,18
44	Male	66	Humerus	Air	600	30	117,07	118,51	109,02	45,93	1,89	5,39
45	Male	66	Humerus	Air	700	10	122,45	129,91	122,94	50,11	0,06	3,03
46	Male	66	Humerus	Air	700	10	141,09	147,71	140,38	58,40	0,20	3,26
47	Male	66	Humerus	Air	700	20	219,89	229,87	223,53	87,05	-0,71	2,65
48	Male	66	Humerus	Air	700	20	222,15	231,00	223,94	87,49	-0,52	2,99
49	Male	66	Humerus	Air	700	30	236,81	242,60	234,04	91,87	0,30	4,20
50	Male	66	Humerus	Air	700	30	239,90	245,94	237,71	92,99	0,32	4,05
51	Male	66	Humerus	Air	800	10	193,18	205,37	199,90	78,76	-1,22	1,96
52	Male	66	Humerus	Air	800	10	218,45	224,05	215,72	85,79	0,40	4,08
53	Male	66	Humerus	Air	800	20	225,88	229,79	220,24	88,32	0,75	4,91
54	Male	66	Humerus	Air	800	20	234,55	239,30	229,96	90,41	0,50	4,71
55	Male	66	Humerus	Air	900	10	223,56	229,77	220,89	83,39	0,09	4,31
56	Male	66	Humerus	Air	900	10	225,99	232,12	223,65	87,83	0,24	4,27
57	Male	66	Humerus	Fat	450	30	53,24	59,60	51,77	19,71	0,51	4,00
58	Male	66	Humerus	Fat	450	30	54,82	61,32	53,50	20,60	0,36	3,78
59	Male	66	Radius	Air	0	0	120,70	99,85	77,75	40,41	9,11	16,25
60	Male	66	Radius	Air	0	0	122,40	101,56	79,28	40,80	8,95	16,28
61	Male	66	Radius	Air	0	0	169,48	138,86	112,44	56,92	11,61	19,27
62	Male	66	Radius	Fat	0	0	169,31	139,22	112,86	57,16	11,42	19,19
63	Male	66	Radius	Air	100	10	195,52	174,29	146,93	68,94	7,36	17,79
64	Male	66	Radius	Air	100	10	196,16	175,71	149,04	69,60	7,24	17,44
65	Male	66	Radius	Air	100	10	169,99	137,20	111,02	56,94	12,42	19,58
66	Male	66	Radius	Air	100	10	171,99	142,62	116,54	57,69	11,24	18,94
67	Male	66	Radius	Air	100	20	190,97	171,09	142,20	67,64	6,70	18,54
68	Male	66	Radius	Air	100	20	192,65	172,73	144,34	68,26	6,56	18,03
69	Male	66	Radius	Air	100	20	177,08	149,77	119,71	60,67	9,26	20,33
70	Male	66	Radius	Air	100	20	177,13	149,59	120,18	60,69	9,61	20,09
71	Male	66	Radius	Air	100	30	194,53	174,80	146,59	69,01	6,61	17,91
72	Male	66	Radius	Air	100	30	199,52	180,64	152,55	71,21	6,35	17,84
73	Male	66	Radius	Air	100	30	170,14	144,65	117,81	58,38	9,53	18,63
74	Male	66	Radius	Air	100	30	174,80	153,08	125,27	61,21	7,66	18,40
75	Male	66	Radius	Air	150	10	180,67	161,04	133,47	63,77	6,83	17,97
76	Male	66	Radius	Air	150	10	192,10	173,40	144,83	68,28	6,83	17,95
77	Male	66	Radius	Air	150	10	184,61	161,44	132,35	64,19	7,91	19,14
78	Male	66	Radius	Air	150	10	185,42	163,50	133,82	65,28	7,39	19,22
79	Male	66	Radius	Air	150	20	190,38	170,15	143,37	67,38	6,95	17,17
80	Male	66	Radius	Air	150	20	193,77	173,00	144,89	68,38	6,92	17,91
81	Male	66	Radius	Air	150	20	175,59	153,51	124,00	60,72	7,69	19,46
82	Male	66	Radius	Air	150	20	177,97	157,03	127,06	62,72	6,80	18,91
83	Male	66	Radius	Air	150	30	181,55	161,79	135,97	64,75	7,18	17,00
84	Male	66	Radius	Air	150	30	185,16	167,16	142,00	66,37	6,53	16,26

Table s9. Raw data of the learning set (3/20)

ID nr	Sex	Age	Skeletal element	Medium	Temp. (°C)	Time (min.)	R	G	B	L*	A*	B*
85	Male	66	Radius	Air	150	30	171,58	152,66	123,95	60,44	6,49	18,58
86	Male	66	Radius	Air	150	30	173,72	155,14	126,78	61,85	6,30	18,15
87	Male	66	Radius	Air	200	10	174,19	156,07	130,14	62,53	6,57	16,91
88	Male	66	Radius	Air	200	10	180,26	162,19	135,95	64,03	6,36	16,77
89	Male	66	Radius	Air	200	10	169,96	155,64	125,33	60,65	3,91	17,75
90	Male	66	Radius	Air	200	10	176,61	163,21	134,31	64,09	4,03	17,46
91	Male	66	Radius	Air	200	20	165,55	146,43	121,34	58,12	7,34	16,80
92	Male	66	Radius	Air	200	20	171,17	153,05	128,05	61,13	6,66	16,36
93	Male	66	Radius	Air	200	20	135,53	130,73	108,08	45,82	3,67	15,52
94	Male	66	Radius	Air	200	20	148,12	143,64	120,83	49,51	3,37	15,69
95	Male	66	Radius	Air	200	30	171,11	147,30	120,11	58,29	8,90	18,61
96	Male	66	Radius	Air	200	30	173,66	150,92	123,50	60,14	8,40	18,61
97	Male	66	Radius	Air	200	30	175,65	159,01	129,10	62,32	5,40	18,42
98	Male	66	Radius	Air	200	30	179,92	166,72	139,55	64,46	4,36	16,52
99	Male	66	Radius	Air	250	10	181,47	154,62	124,38	61,04	10,15	21,07
100	Male	66	Radius	Air	250	10	184,93	157,70	126,72	63,04	9,93	21,47
101	Male	66	Radius	Air	250	10	177,45	162,93	135,22	63,77	4,76	17,05
102	Male	66	Radius	Air	250	10	184,67	171,08	143,43	66,60	4,30	16,54
103	Male	66	Radius	Air	250	20	164,64	129,21	99,66	54,46	13,69	22,31
104	Male	66	Radius	Air	250	20	172,46	137,87	108,18	56,17	13,14	21,72
105	Male	66	Radius	Air	250	20	142,11	111,04	83,89	47,75	13,32	21,66
106	Male	66	Radius	Air	250	20	139,95	108,43	82,37	38,12	11,61	17,09
107	Male	66	Radius	Air	250	30	141,41	102,38	77,33	43,66	16,93	21,65
108	Male	66	Radius	Air	250	30	145,44	105,90	80,09	45,91	17,10	22,50
109	Male	66	Radius	Air	250	30	136,45	98,18	74,43	40,66	16,42	20,17
110	Male	66	Radius	Air	250	30	143,57	102,37	76,46	43,28	17,39	22,06
111	Male	66	Radius	Air	300	10	156,54	115,50	87,11	47,50	16,81	22,25
112	Male	66	Radius	Air	300	10	158,20	119,22	90,55	47,08	15,96	21,68
113	Male	66	Radius	Air	300	10	150,02	110,16	84,22	46,49	16,74	21,70
114	Male	66	Radius	Air	300	10	167,30	125,33	95,11	52,25	16,32	23,59
115	Male	66	Radius	Air	300	20	106,70	86,65	69,62	34,15	10,04	13,43
116	Male	66	Radius	Air	300	20	111,25	90,73	74,38	35,92	10,44	13,28
117	Male	66	Radius	Air	300	20	61,84	64,51	56,21	22,73	1,67	4,50
118	Male	66	Radius	Air	300	20	66,57	65,45	56,24	24,65	4,06	6,52
119	Male	66	Radius	Air	300	30	70,82	64,20	52,81	23,50	5,21	7,93
120	Male	66	Radius	Air	300	30	72,49	66,24	54,94	24,20	5,29	8,10
121	Male	66	Radius	Air	300	30	51,03	57,60	50,15	19,39	0,43	3,49
122	Male	66	Radius	Air	300	30	61,50	67,43	60,00	23,42	0,61	3,54
123	Male	66	Radius	Air	350	10	73,22	68,11	58,34	25,24	5,01	6,94
124	Male	66	Radius	Air	350	10	77,56	70,49	58,77	26,22	5,36	8,28
125	Male	66	Radius	Air	350	10	59,94	62,66	53,91	21,78	1,88	6,13
126	Male	66	Radius	Air	350	10	61,05	61,44	52,48	21,37	2,73	5,50
127	Male	66	Radius	Air	350	20	53,18	60,13	52,77	19,94	0,15	3,35

Table s9. Raw data of the learning set (4/20)

ID nr	Sex	Age	Skeletal element	Medium	Temp. (°C)	Time (min.)	R	G	B	L*	A*	B*
128	Male	66	Radius	Air	350	20	53,76	59,95	52,59	20,07	0,54	3,57
129	Male	66	Radius	Air	350	20	66,62	73,89	66,38	26,61	-0,02	20,27
130	Male	66	Radius	Air	350	20	68,01	74,87	67,13	27,06	0,14	3,64
131	Male	66	Radius	Air	350	30	49,48	55,17	47,08	17,98	0,58	3,89
132	Male	66	Radius	Air	350	30	53,63	59,84	52,17	19,36	0,39	3,74
133	Male	66	Radius	Air	350	30	63,73	71,59	64,29	25,35	-0,16	3,23
134	Male	66	Radius	Air	350	30	78,81	87,16	79,98	32,21	-0,35	3,07
135	Male	66	Radius	Air	400	10	54,41	61,89	54,55	20,59	-0,04	3,34
136	Male	66	Radius	Air	400	10	59,25	66,38	58,79	23,13	0,06	3,54
137	Male	66	Radius	Air	400	10	63,69	71,24	63,57	25,17	-0,11	3,49
138	Male	66	Radius	Air	400	10	65,72	73,84	66,29	26,56	-0,13	3,30
139	Male	66	Radius	Air	400	20	46,13	53,05	45,37	16,57	0,06	3,44
140	Male	66	Radius	Air	400	20	50,21	57,18	49,60	19,36	0,17	3,45
141	Male	66	Radius	Air	400	20	67,85	76,00	68,56	27,23	-0,33	3,28
142	Male	66	Radius	Air	400	20	69,38	77,57	70,08	27,70	-0,25	3,23
143	Male	66	Radius	Fat	100	10	169,58	148,65	123,42	58,94	7,90	16,97
144	Male	66	Radius	Fat	100	10	173,50	150,60	124,04	59,62	8,55	17,96
145	Male	66	Radius	Fat	100	10	186,15	162,06	134,68	64,80	8,67	18,43
146	Male	66	Radius	Fat	100	10	190,58	167,71	141,02	66,96	8,15	17,77
147	Male	66	Radius	Fat	100	20	192,75	168,70	137,50	67,42	7,95	20,31
148	Male	66	Radius	Fat	100	20	199,09	175,53	144,41	69,08	6,53	19,72
149	Male	66	Radius	Fat	100	20	178,17	154,02	128,06	61,66	8,67	17,63
150	Male	66	Radius	Fat	100	20	184,08	162,31	136,70	64,31	8,06	16,09
151	Male	66	Radius	Fat	100	30	182,17	159,91	131,86	64,54	7,64	18,31
152	Male	66	Radius	Fat	100	30	183,83	162,96	135,40	64,43	7,27	17,87
153	Male	66	Radius	Fat	100	30	180,25	164,27	139,39	63,91	5,73	15,52
154	Male	66	Radius	Fat	100	30	181,72	160,54	132,73	64,53	7,22	18,22
155	Male	66	Radius	Fat	150	10	170,75	151,30	125,23	59,84	7,14	17,21
156	Male	66	Radius	Fat	150	10	170,86	151,20	124,99	60,72	7,11	17,33
157	Male	66	Radius	Fat	150	10	174,84	160,31	136,45	63,20	5,40	15,13
158	Male	66	Radius	Fat	150	10	191,47	173,56	147,18	68,90	5,95	16,59
159	Male	66	Radius	Fat	150	20	160,69	142,40	116,35	56,94	6,83	17,33
160	Male	66	Radius	Fat	150	20	162,46	143,98	117,65	57,38	6,88	17,41
161	Male	66	Radius	Fat	150	20	186,58	168,08	140,90	66,72	6,40	17,48
162	Male	66	Radius	Fat	150	20	187,57	168,01	139,85	66,02	6,62	17,62
163	Male	66	Radius	Fat	150	30	145,95	128,10	102,62	52,48	6,69	17,15
164	Male	66	Radius	Fat	150	30	148,76	130,65	104,74	52,68	6,96	17,15
165	Male	66	Radius	Fat	150	30	177,20	162,17	137,84	64,02	5,60	15,59
166	Male	66	Radius	Fat	150	30	178,71	164,05	139,91	65,04	5,27	15,18
167	Male	66	Radius	Fat	200	10	174,51	153,36	125,85	61,01	7,49	18,04
168	Male	66	Radius	Fat	200	10	196,84	175,01	143,28	69,55	6,80	19,95
169	Male	66	Radius	Fat	200	10	187,48	172,17	146,83	67,98	5,31	15,87

Table s9. Raw data of the learning set (5/20)

ID nr	Sex	Age	Skeletal element	Medium	Temp. (°C)	Time (min.)	R	G	B	L*	A*	B*
170	Male	66	Radius	Fat	200	10	193,82	176,62	149,20	69,93	5,60	16,54
171	Male	66	Radius	Fat	200	20	169,80	146,80	119,37	59,28	8,24	18,40
172	Male	66	Radius	Fat	200	20	174,71	152,61	125,20	60,59	8,01	18,25
173	Male	66	Radius	Fat	200	20	186,11	169,28	142,76	66,94	5,77	16,71
174	Male	66	Radius	Fat	200	20	191,74	176,61	151,39	69,16	5,13	15,53
175	Male	66	Radius	Fat	200	30	174,98	148,82	119,59	60,85	9,13	19,83
176	Male	66	Radius	Fat	200	30	179,40	155,82	127,18	62,43	8,21	18,96
177	Male	66	Radius	Fat	200	30	189,43	170,92	143,47	68,15	6,34	17,60
178	Male	66	Radius	Fat	200	30	192,10	175,28	149,00	68,71	5,78	16,46
179	Male	66	Radius	Fat	250	10	179,57	157,95	129,03	63,55	7,49	18,95
180	Male	66	Radius	Fat	250	10	183,04	160,65	132,01	64,21	7,68	18,67
181	Male	66	Radius	Fat	250	10	200,83	183,65	156,14	71,54	5,64	17,00
182	Male	66	Radius	Fat	250	10	201,01	183,73	156,33	72,10	5,45	16,74
183	Male	66	Radius	Fat	250	20	162,00	136,88	107,92	55,47	9,09	19,83
184	Male	66	Radius	Fat	250	20	166,62	139,94	110,71	56,43	9,70	20,16
185	Male	66	Radius	Fat	250	20	184,59	167,91	140,84	68,36	5,48	17,42
186	Male	66	Radius	Fat	250	20	188,44	172,80	145,28	67,83	5,09	16,87
187	Male	66	Radius	Fat	250	30	164,51	125,30	93,22	52,40	14,89	24,17
188	Male	66	Radius	Fat	250	30	165,67	127,39	95,23	53,33	14,67	24,01
189	Male	66	Radius	Fat	250	30	177,62	142,49	109,44	58,41	12,75	23,65
190	Male	66	Radius	Fat	250	30	178,64	147,26	114,96	59,52	11,03	22,30
191	Male	66	Radius	Fat	300	10	177,29	152,59	122,86	60,96	8,59	19,74
192	Male	66	Radius	Fat	300	10	178,29	153,01	122,97	61,40	8,71	19,99
193	Male	66	Radius	Fat	300	10	177,31	139,41	106,70	57,08	13,91	23,91
194	Male	66	Radius	Fat	300	10	181,66	143,81	110,61	59,22	13,60	23,91
195	Male	66	Radius	Fat	300	20	129,39	98,05	76,00	40,36	13,87	18,24
196	Male	66	Radius	Fat	300	20	125,33	93,98	72,58	39,01	14,10	18,10
197	Male	66	Radius	Fat	300	20	75,99	71,61	59,91	26,60	4,05	7,76
198	Male	66	Radius	Fat	300	20	78,93	74,76	62,77	26,73	4,00	7,96
199	Male	66	Radius	Fat	300	30	63,27	63,44	53,20	22,41	2,28	6,01
200	Male	66	Radius	Fat	300	30	66,24	65,61	54,75	23,74	2,76	6,80
201	Male	66	Radius	Fat	300	30	57,12	62,63	54,69	21,19	0,74	3,96
202	Male	66	Radius	Fat	300	30	58,25	64,71	57,13	21,83	0,45	3,75
203	Male	66	Radius	Fat	350	10	104,91	90,86	73,86	35,99	7,29	12,49
204	Male	66	Radius	Fat	350	10	93,64	79,74	65,57	31,04	7,91	10,90
205	Male	66	Radius	Fat	350	10	100,94	78,61	61,20	31,45	11,13	14,42
206	Male	66	Radius	Fat	350	10	101,70	79,76	62,42	32,25	11,21	14,55
207	Male	66	Radius	Fat	350	20	74,05	73,83	62,27	27,15	2,44	7,15
208	Male	66	Radius	Fat	350	20	86,39	82,08	68,20	30,91	3,77	8,94
209	Male	66	Radius	Fat	350	20	59,50	64,06	55,39	22,04	0,88	4,49
210	Male	66	Radius	Fat	350	20	59,95	63,56	54,57	22,76	1,56	5,34
211	Male	66	Radius	Fat	350	30	54,06	61,06	53,88	20,48	0,19	3,16
212	Male	66	Radius	Fat	350	30	56,32	63,17	55,90	21,70	0,32	3,28

Table s9. Raw data of the learning set (6/20)

ID nr	Sex	Age	Skeletal element	Medium	Temp. (°C)	Time (min.)	R	G	B	L*	A*	B*
213	Male	66	Radius	Fat	350	30	60,04	64,18	55,28	22,56	1,07	4,78
214	Male	66	Radius	Fat	350	30	60,19	64,10	55,39	21,60	1,20	4,83
215	Male	66	Radius	Fat	400	10	61,25	63,57	54,79	21,74	1,84	5,01
216	Male	66	Radius	Fat	400	10	64,81	64,59	55,04	22,62	2,63	5,69
217	Male	66	Radius	Fat	400	10	59,38	64,10	55,59	22,27	0,87	4,41
218	Male	66	Radius	Fat	400	10	59,70	63,53	54,51	22,28	1,18	4,89
219	Male	66	Radius	Fat	400	20	51,70	58,34	51,20	19,13	0,44	3,26
220	Male	66	Radius	Fat	400	20	53,96	60,26	52,79	20,10	0,58	3,61
221	Male	66	Radius	Fat	400	20	56,36	63,64	56,59	21,74	0,07	3,17
222	Male	66	Radius	Fat	400	20	57,04	63,85	56,60	22,14	0,24	3,33
223	Male	66	Radius	Air	450	30	48,35	54,09	45,99	18,28	0,56	3,99
224	Male	66	Radius	Air	450	30	53,41	60,23	52,67	19,98	0,21	3,54
225	Male	66	Radius	Air	500	10	64,88	68,81	60,06	23,81	0,97	4,47
226	Male	66	Radius	Air	500	10	90,44	90,27	80,08	34,40	2,58	6,15
227	Male	66	Radius	Air	500	20	124,21	119,87	108,37	46,60	3,84	7,40
228	Male	66	Radius	Air	500	20	122,10	120,64	110,09	46,63	2,87	6,42
229	Male	66	Radius	Air	500	30	121,06	118,22	106,99	46,29	3,31	7,14
230	Male	66	Radius	Air	500	30	121,77	117,64	106,32	46,14	3,76	7,29
231	Male	66	Radius	Air	600	10	101,18	102,20	92,80	38,37	2,14	5,44
232	Male	66	Radius	Air	600	10	107,07	107,22	96,98	40,52	2,37	6,03
233	Male	66	Radius	Air	600	20	105,96	107,82	98,86	41,60	1,96	5,10
234	Male	66	Radius	Air	600	20	106,09	107,71	98,59	40,85	1,90	5,13
235	Male	66	Radius	Air	600	30	110,42	112,30	103,13	43,28	1,80	5,16
236	Male	66	Radius	Air	600	30	118,54	119,23	109,30	44,42	2,16	5,75
237	Male	66	Radius	Air	700	10	98,35	103,23	95,47	37,74	0,87	3,77
238	Male	66	Radius	Air	700	10	108,05	113,06	105,04	41,86	0,95	4,22
239	Male	66	Radius	Air	700	20	191,33	200,94	195,47	76,36	-0,46	2,14
240	Male	66	Radius	Air	700	20	198,14	207,04	200,88	78,70	-0,35	2,67
241	Male	66	Radius	Air	700	30	241,64	248,42	240,65	93,27	0,13	3,63
242	Male	66	Radius	Air	700	30	246,72	254,12	246,64	96,27	-0,02	3,47
243	Male	66	Radius	Air	800	10	221,64	227,61	219,11	85,33	0,39	4,17
244	Male	66	Radius	Air	800	10	223,22	229,33	221,29	87,11	0,28	3,93
245	Male	66	Radius	Air	800	20	241,48	247,37	238,58	93,04	0,21	4,34
246	Male	66	Radius	Air	800	20	243,26	247,57	237,34	93,78	0,57	5,37
247	Male	66	Radius	Air	900	10	241,16	247,30	239,08	94,75	0,30	4,08
248	Male	66	Radius	Air	900	10	247,35	253,53	245,21	96,85	0,21	4,11
249	Male	66	Radius	Fat	450	30	72,91	79,68	72,04	29,09	0,30	3,53
250	Male	66	Radius	Fat	450	30	76,24	84,44	77,57	30,51	-0,09	3,02
251	Male	66	Ulna	Air	0	0	156,85	137,39	112,25	54,89	7,38	16,88
252	Male	66	Ulna	Fat	0	0	156,61	134,50	108,75	54,20	8,72	18,10
253	Male	66	Ulna	Air	0	0	182,44	152,41	126,16	61,94	11,24	18,93
254	Male	66	Ulna	Fat	0	0	188,47	158,64	132,10	65,22	10,44	17,52
255	Male	66	Ulna	Air	100	10	135,91	119,12	94,64	47,61	7,59	17,15

Table s9. Raw data of the learning set (7/20)

ID nr	Sex	Age	Skeletal element	Medium	Temp. (°C)	Time (min.)	R	G	B	L*	A*	B*
256	Male	66	Ulna	Air	100	10	136,36	117,23	92,53	46,82	7,68	17,13
257	Male	66	Ulna	Air	100	10	186,50	156,57	127,35	62,73	10,70	20,41
258	Male	66	Ulna	Air	100	10	186,66	159,51	130,94	63,48	9,79	19,59
259	Male	66	Ulna	Air	100	20	148,56	131,39	108,60	53,54	9,91	15,54
260	Male	66	Ulna	Air	100	20	148,71	129,05	105,04	51,33	7,83	16,55
261	Male	66	Ulna	Air	100	20	194,14	166,38	136,29	66,51	9,33	19,94
262	Male	66	Ulna	Air	100	20	194,67	169,11	139,17	67,77	8,59	19,71
263	Male	66	Ulna	Air	100	30	146,19	128,30	105,02	52,90	7,05	16,04
264	Male	66	Ulna	Air	100	30	155,71	138,35	114,38	56,26	6,48	15,83
265	Male	66	Ulna	Air	100	30	177,87	154,57	126,17	62,56	7,86	18,50
266	Male	66	Ulna	Air	100	30	182,35	157,78	130,59	63,30	8,84	18,42
267	Male	66	Ulna	Air	150	10	159,32	140,38	116,17	56,35	7,32	16,29
268	Male	66	Ulna	Air	150	10	161,89	143,57	119,10	57,74	6,90	16,32
269	Male	66	Ulna	Air	150	10	174,27	153,28	125,27	61,33	7,33	18,44
270	Male	66	Ulna	Air	150	10	174,72	154,58	126,14	61,87	6,90	18,32
271	Male	66	Ulna	Air	150	20	134,52	134,09	109,08	54,30	7,43	16,84
272	Male	66	Ulna	Air	150	20	159,98	142,34	118,02	57,03	6,71	16,09
273	Male	66	Ulna	Air	150	20	177,03	156,20	127,63	62,08	7,26	18,71
274	Male	66	Ulna	Air	150	20	178,02	157,86	129,55	62,95	7,04	18,59
275	Male	66	Ulna	Air	150	30	153,45	138,12	114,84	56,48	5,81	15,18
276	Male	66	Ulna	Air	150	30	155,81	139,90	116,28	55,95	6,28	15,73
277	Male	66	Ulna	Air	150	30	163,26	143,69	115,69	57,34	6,96	18,31
278	Male	66	Ulna	Air	150	30	163,32	144,08	116,50	57,48	6,80	17,96
279	Male	66	Ulna	Air	200	10	161,01	142,88	116,71	56,71	6,76	17,38
280	Male	66	Ulna	Air	200	10	163,27	143,93	116,77	57,37	6,89	17,68
281	Male	66	Ulna	Air	200	10	164,38	150,28	121,73	58,81	4,53	17,81
282	Male	66	Ulna	Air	200	10	166,68	152,09	122,40	59,96	4,62	18,39
283	Male	66	Ulna	Air	200	20	168,50	149,25	122,50	60,09	6,76	17,37
284	Male	66	Ulna	Air	200	20	169,29	150,49	124,35	60,08	6,80	17,07
285	Male	66	Ulna	Air	200	20	179,56	165,34	136,64	61,68	4,38	17,47
286	Male	66	Ulna	Air	200	20	180,27	167,10	139,51	65,74	4,23	16,85
287	Male	66	Ulna	Air	200	30	168,33	144,18	115,15	59,15	8,08	19,26
288	Male	66	Ulna	Air	200	30	171,48	146,02	116,00	58,52	9,02	20,15
289	Male	66	Ulna	Air	200	30	162,60	143,41	114,94	57,75	6,98	18,90
290	Male	66	Ulna	Air	200	30	176,97	159,35	130,95	62,65	5,91	17,91
291	Male	66	Ulna	Air	250	10	168,80	159,11	132,78	59,71	3,10	16,07
292	Male	66	Ulna	Air	250	10	170,56	160,96	135,44	61,39	3,29	15,52
293	Male	66	Ulna	Air	250	20	157,12	124,61	93,77	52,31	12,38	23,04
294	Male	66	Ulna	Air	250	20	158,16	126,23	94,94	52,92	12,06	22,71
295	Male	66	Ulna	Air	250	20	150,02	114,27	84,52	48,06	14,52	23,26
296	Male	66	Ulna	Air	250	20	152,35	114,41	84,53	47,25	15,34	23,22
297	Male	66	Ulna	Air	250	30	134,78	97,35	74,12	40,92	16,87	20,48
298	Male	66	Ulna	Air	250	30	139,38	100,67	77,44	43,79	17,34	20,96

Table s9. Raw data of the learning set (8/20)

ID nr	Sex	Age	Skeletal element	Medium	Temp. (°C)	Time (min.)	R	G	B	L*	A*	B*
299	Male	66	Ulna	Air	250	30	112,65	80,94	61,83	33,38	15,02	17,29
300	Male	66	Ulna	Air	250	30	122,41	85,67	64,05	35,22	16,26	18,97
301	Male	66	Ulna	Air	300	10	152,59	109,59	79,12	46,78	17,59	25,10
302	Male	66	Ulna	Air	300	10	159,93	118,86	86,85	51,39	15,47	24,91
303	Male	66	Ulna	Air	300	10	138,12	103,70	76,35	42,73	14,28	21,32
304	Male	66	Ulna	Air	300	10	117,10	83,48	63,04	35,40	15,79	18,50
305	Male	66	Ulna	Air	300	20	80,38	69,03	56,54	26,54	7,54	9,96
306	Male	66	Ulna	Air	300	20	75,94	67,49	56,39	26,68	6,44	8,70
307	Male	66	Ulna	Air	300	20	51,87	57,73	50,08	19,72	0,73	3,80
308	Male	66	Ulna	Air	300	20	54,66	59,80	52,01	20,64	1,01	3,89
309	Male	66	Ulna	Air	300	30	58,97	59,75	50,93	20,81	2,71	5,43
310	Male	66	Ulna	Air	300	30	60,28	60,25	51,05	21,61	3,00	5,88
311	Male	66	Ulna	Air	300	30	56,34	62,14	54,60	21,57	0,69	3,70
312	Male	66	Ulna	Air	300	30	61,57	67,03	59,28	23,40	0,68	3,84
313	Male	66	Ulna	Air	350	10	86,34	75,31	61,92	29,69	6,72	9,95
314	Male	66	Ulna	Air	350	10	86,57	76,14	62,90	29,81	6,43	9,77
315	Male	66	Ulna	Air	350	10	56,19	60,16	51,98	20,38	1,33	4,26
316	Male	66	Ulna	Air	350	10	61,65	63,37	54,79	22,20	2,21	4,98
317	Male	66	Ulna	Air	350	20	51,04	58,08	50,69	19,30	0,10	3,34
318	Male	66	Ulna	Air	350	20	56,68	64,07	56,81	20,85	0,08	3,39
319	Male	66	Ulna	Air	350	20	61,93	69,14	61,41	24,32	-0,06	3,55
320	Male	66	Ulna	Air	350	20	72,46	79,57	71,79	29,12	0,11	3,61
321	Male	66	Ulna	Air	350	30	49,65	56,17	48,37	18,19	0,27	3,74
322	Male	66	Ulna	Air	350	30	51,05	57,64	49,70	19,02	0,14	3,80
323	Male	66	Ulna	Air	350	30	71,15	78,75	71,28	28,41	-0,13	3,38
324	Male	66	Ulna	Air	350	30	72,70	80,69	73,43	29,37	-0,21	3,14
325	Male	66	Ulna	Air	400	10	52,87	59,71	51,94	19,76	0,13	3,65
326	Male	66	Ulna	Air	400	10	56,55	64,03	56,52	21,96	-0,11	3,40
327	Male	66	Ulna	Air	400	10	64,02	71,36	63,76	25,70	-0,04	3,43
328	Male	66	Ulna	Air	400	10	65,14	72,55	64,78	25,90	-0,11	3,56
329	Male	66	Ulna	Air	400	20	53,37	60,09	52,31	17,53	0,08	3,63
330	Male	66	Ulna	Air	400	20	54,94	61,39	53,45	22,95	0,49	4,07
331	Male	66	Ulna	Air	400	20	64,62	72,18	64,40	26,74	-0,30	3,43
332	Male	66	Ulna	Air	400	20	71,37	79,81	72,51	29,44	-0,47	3,08
333	Male	66	Ulna	Fat	100	10	156,92	136,60	110,59	54,56	7,82	17,74
334	Male	66	Ulna	Fat	100	10	163,26	142,13	115,95	57,12	7,99	17,89
335	Male	66	Ulna	Fat	100	10	185,57	160,05	133,58	63,66	9,04	17,98
336	Male	66	Ulna	Fat	100	10	188,31	159,61	133,06	64,30	10,48	18,58
337	Male	66	Ulna	Fat	100	20	153,09	134,86	111,66	53,94	7,11	15,48
338	Male	66	Ulna	Fat	100	20	154,31	132,99	108,79	53,80	8,35	16,78
339	Male	66	Ulna	Fat	100	30	153,27	133,76	109,07	54,10	7,75	17,09
340	Male	66	Ulna	Fat	100	30	159,42	139,73	115,00	57,43	7,32	16,66
341	Male	66	Ulna	Fat	100	20	175,96	154,70	127,08	61,74	7,51	18,23

Table s9. Raw data of the learning set (9/20)

ID nr	Sex	Age	Skeletal element	Medium	Temp. (°C)	Time (min.)	R	G	B	L*	A*	B*
342	Male	66	Ulna	Fat	100	20	180,25	159,19	131,28	63,36	7,30	18,22
343	Male	66	Ulna	Fat	150	10	154,06	134,26	107,42	53,38	7,44	17,97
344	Male	66	Ulna	Fat	150	10	154,26	134,82	108,07	54,55	7,19	18,01
345	Male	66	Ulna	Fat	100	30	177,80	159,50	132,27	64,88	5,92	17,53
346	Male	66	Ulna	Fat	100	30	183,87	164,34	135,82	65,34	6,64	18,38
347	Male	66	Ulna	Fat	150	20	146,26	127,36	102,57	50,84	7,40	16,91
348	Male	66	Ulna	Fat	150	20	161,69	144,33	119,82	57,94	6,53	16,13
349	Male	66	Ulna	Fat	150	10	188,08	172,03	146,69	68,93	5,46	16,26
350	Male	66	Ulna	Fat	150	10	189,45	170,62	142,82	67,83	6,06	17,55
351	Male	66	Ulna	Fat	150	30	145,41	127,10	103,20	51,00	7,23	16,22
352	Male	66	Ulna	Fat	150	30	148,50	130,46	106,32	51,89	7,17	16,31
353	Male	66	Ulna	Fat	150	20	166,55	152,82	128,95	60,35	5,14	15,19
354	Male	66	Ulna	Fat	150	20	169,82	157,78	136,05	58,51	5,87	15,48
355	Male	66	Ulna	Fat	150	30	182,94	166,62	140,83	66,20	5,64	16,30
356	Male	66	Ulna	Fat	150	30	186,15	171,67	147,51	68,24	5,17	15,27
357	Male	66	Ulna	Fat	200	10	161,21	142,19	116,68	56,80	7,27	17,21
358	Male	66	Ulna	Fat	200	10	162,89	142,91	116,36	58,01	7,29	17,85
359	Male	66	Ulna	Fat	200	20	156,12	136,89	112,41	54,68	7,49	16,56
360	Male	66	Ulna	Fat	200	20	157,56	137,15	112,27	55,06	7,78	16,90
361	Male	66	Ulna	Fat	200	10	171,85	157,31	130,13	61,03	4,71	16,28
362	Male	66	Ulna	Fat	200	10	172,95	156,62	128,04	61,92	5,18	17,44
363	Male	66	Ulna	Fat	200	30	161,27	142,42	116,61	57,36	7,07	17,22
364	Male	66	Ulna	Fat	200	30	161,34	141,74	116,13	56,84	7,46	17,30
365	Male	66	Ulna	Fat	200	20	169,23	156,44	128,52	62,24	4,07	17,13
366	Male	66	Ulna	Fat	200	20	171,23	158,19	129,56	64,30	3,93	17,36
367	Male	66	Ulna	Fat	200	30	171,79	155,58	128,54	63,30	5,20	16,79
368	Male	66	Ulna	Fat	200	30	176,31	160,58	132,99	63,65	5,09	16,90
369	Male	66	Ulna	Fat	250	10	160,91	136,51	109,40	57,86	8,16	18,21
370	Male	66	Ulna	Fat	250	10	167,26	143,98	116,20	58,51	8,25	18,88
371	Male	66	Ulna	Fat	250	20	119,46	128,26	101,32	52,28	7,64	18,53
372	Male	66	Ulna	Fat	250	20	147,28	123,87	96,63	51,98	8,12	18,70
373	Male	66	Ulna	Fat	250	10	192,80	178,70	154,17	71,52	5,01	15,16
374	Male	66	Ulna	Fat	250	10	193,86	179,07	154,19	70,33	5,08	15,33
375	Male	66	Ulna	Fat	250	30	161,38	126,08	94,14	52,07	13,38	23,54
376	Male	66	Ulna	Fat	250	30	161,85	127,31	95,97	52,74	13,18	23,24
377	Male	66	Ulna	Fat	250	20	180,68	148,21	116,69	60,33	11,79	22,38
378	Male	66	Ulna	Fat	250	20	183,11	152,25	120,74	63,62	11,53	23,26
379	Male	66	Ulna	Fat	300	10	161,31	131,19	100,23	54,78	10,71	22,01
380	Male	66	Ulna	Fat	300	10	161,85	129,61	98,46	54,55	10,46	21,47
381	Male	66	Ulna	Fat	250	30	146,12	108,19	80,16	45,27	15,33	22,19
382	Male	66	Ulna	Fat	250	30	149,16	111,68	83,00	46,71	14,30	22,77
383	Male	66	Ulna	Fat	300	20	113,49	86,71	69,22	35,45	12,85	14,88
384	Male	66	Ulna	Fat	300	20	114,84	86,24	67,72	35,39	13,53	15,89

Table s9. Raw data of the learning set (10/20)

ID nr	Sex	Age	Skeletal element	Medium	Temp. (°C)	Time (min.)	R	G	B	L*	A*	B*
385	Male	66	Ulna	Fat	300	10	159,41	121,88	91,07	50,54	14,60	23,41
386	Male	66	Ulna	Fat	300	10	163,65	126,13	95,32	52,85	14,75	23,61
387	Male	66	Ulna	Fat	300	30	59,38	64,08	55,90	21,81	1,61	4,83
388	Male	66	Ulna	Fat	300	30	61,45	63,49	54,72	22,63	1,89	4,94
389	Male	66	Ulna	Fat	300	20	67,89	68,60	59,08	24,53	2,50	5,80
390	Male	66	Ulna	Fat	300	20	68,69	68,77	58,87	24,89	2,66	6,09
391	Male	66	Ulna	Fat	350	10	94,01	77,36	62,71	30,41	9,29	11,86
392	Male	66	Ulna	Fat	350	10	80,95	69,68	57,26	26,41	7,14	9,47
393	Male	66	Ulna	Fat	300	30	61,47	66,89	59,01	23,69	0,83	4,00
394	Male	66	Ulna	Fat	300	30	62,67	68,30	60,64	24,04	0,64	3,74
395	Male	66	Ulna	Fat	350	20	62,52	63,87	54,11	22,31	2,05	5,64
396	Male	66	Ulna	Fat	350	20	67,25	67,34	56,61	24,85	2,59	6,76
397	Male	66	Ulna	Fat	350	10	162,41	133,48	102,49	53,89	10,56	21,62
398	Male	66	Ulna	Fat	350	10	166,91	140,84	110,22	57,20	9,23	20,92
399	Male	66	Ulna	Fat	350	30	63,30	66,94	58,19	23,66	1,51	4,88
400	Male	66	Ulna	Fat	350	30	67,19	69,24	59,42	24,94	1,86	5,69
401	Male	66	Ulna	Fat	350	20	57,52	61,77	53,58	21,57	1,21	4,43
402	Male	66	Ulna	Fat	350	20	59,31	63,56	55,16	22,08	1,04	4,38
403	Male	66	Ulna	Fat	400	10	66,54	64,97	53,98	23,35	3,37	7,19
404	Male	66	Ulna	Fat	400	10	73,50	68,27	56,05	24,98	4,69	8,43
405	Male	66	Ulna	Fat	350	30	54,42	59,24	51,07	20,34	0,98	4,26
406	Male	66	Ulna	Fat	350	30	55,82	60,31	52,04	20,65	1,05	4,30
407	Male	66	Ulna	Fat	400	20	59,45	64,22	55,99	22,00	0,98	4,38
408	Male	66	Ulna	Fat	400	20	61,90	64,64	55,38	22,43	1,77	5,34
409	Male	66	Ulna	Fat	400	10	53,94	59,52	51,35	20,42	0,65	4,18
410	Male	66	Ulna	Fat	400	10	54,11	59,94	51,99	20,43	0,50	3,87
411	Male	66	Ulna	Fat	400	20	59,99	65,53	57,33	22,36	0,54	4,04
412	Male	66	Ulna	Fat	400	20	62,90	67,62	58,97	23,70	0,85	4,48
413	Male	56	Radius	Air	800	30	249,38	242,83	238,12	93,21	0,38	3,91
414	Male	56	Radius	Air	800	30	252,67	245,31	241,77	94,12	0,33	3,51
415	Male	56	Radius	Air	800	30	254,88	247,87	244,44	96,32	0,40	4,37
416	Male	56	Radius	Air	800	30	255,22	248,30	244,76	96,45	0,08	4,24
417	Male	56	Radius	Air	800	30	254,88	247,84	244,34	96,10	0,53	4,05
418	Male	56	Radius	Air	800	30	241,45	234,74	232,38	93,12	0,09	3,93
419	Male	56	Radius	Air	800	30	242,60	235,65	232,77	95,45	0,45	3,79
420	Male	56	Radius	Air	800	30	252,78	244,57	242,87	96,88	0,23	3,38
421	Male	56	Radius	Air	800	30	253,93	246,96	243,32	95,42	0,32	5,22
422	Male	56	Radius	Air	800	30	250,55	243,83	239,77	96,13	-0,17	4,10
423	Male	56	Radius	Air	900	20	254,22	256,97	254,13	93,77	0,22	2,86
424	Male	56	Radius	Air	900	20	254,34	245,74	243,11	94,35	0,33	4,34
425	Male	56	Radius	Air	900	20	255,99	249,21	245,67	95,33	0,14	3,29
426	Male	56	Radius	Air	900	20	251,66	245,30	241,76	91,31	0,14	4,24
427	Male	56	Radius	Air	900	20	253,76	246,10	244,12	92,89	0,41	4,36

Table s9. Raw data of the learning set (11/20)

ID nr	Sex	Age	Skeletal element	Medium	Temp. (°C)	Time (min.)	R	G	B	L*	A*	B*
428	Male	56	Radius	Air	900	20	249,77	242,78	240,08	93,46	0,05	4,05
429	Male	56	Radius	Air	900	20	252,24	245,42	241,76	92,96	0,31	3,99
430	Male	56	Radius	Air	900	20	250,64	243,76	241,77	92,24	0,54	3,87
431	Male	56	Radius	Air	900	20	250,31	243,52	240,76	95,54	0,12	5,68
432	Male	56	Radius	Air	900	20	252,97	245,41	243,06	92,35	0,01	3,79
433	Male	56	Ulna	Air	800	30	246,87	239,67	236,11	93,83	0,40	4,50
434	Male	56	Ulna	Air	800	30	244,99	238,24	234,68	92,87	0,46	4,05
435	Male	56	Ulna	Air	800	30	255,60	248,32	245,81	91,76	0,13	3,68
436	Male	56	Ulna	Air	800	30	253,65	246,64	243,73	90,80	0,77	4,31
437	Male	56	Ulna	Air	800	30	252,55	245,57	242,43	94,88	0,77	3,38
438	Male	56	Ulna	Air	800	30	252,85	245,31	241,54	93,92	1,19	2,53
439	Male	56	Ulna	Air	800	30	254,40	247,44	244,77	93,49	-0,12	4,91
440	Male	56	Ulna	Air	800	30	249,70	242,76	239,77	92,21	0,13	4,01
441	Male	56	Ulna	Air	800	30	248,34	241,85	238,79	93,87	0,09	2,61
442	Male	56	Ulna	Air	800	30	253,34	246,30	239,89	94,21	0,45	2,70
443	Male	56	Ulna	Air	900	30	254,43	246,32	244,67	94,56	0,41	3,68
444	Male	56	Ulna	Air	900	30	255,51	248,72	246,17	94,75	0,35	4,27
445	Male	56	Ulna	Air	450	20	74,93	82,31	77,13	23,98	-0,39	2,95
446	Male	56	Ulna	Air	900	30	252,36	245,31	242,82	91,08	0,04	4,09
447	Male	56	Ulna	Air	450	20	76,93	84,68	79,29	27,97	0,43	3,26
448	Male	56	Ulna	Air	900	30	249,74	243,74	240,15	94,88	0,12	3,28
449	Male	56	Ulna	Air	900	30	252,90	246,10	242,91	94,54	0,11	3,91
450	Male	56	Ulna	Air	450	20	75,93	83,70	79,06	21,98	0,66	3,49
451	Male	56	Ulna	Air	900	30	252,52	245,48	243,06	92,32	0,06	4,23
452	Male	56	Ulna	Air	450	20	77,93	85,74	80,02	25,97	-0,53	3,35
453	Male	56	Ulna	Air	900	30	254,81	247,57	245,51	95,00	0,61	4,34
454	Male	56	Ulna	Air	450	20	69,94	77,55	72,48	25,97	0,27	3,16
455	Male	56	Ulna	Air	900	30	253,11	246,32	243,81	92,75	0,23	4,07
456	Male	56	Ulna	Air	450	20	62,94	70,62	65,86	25,97	-0,04	3,47
457	Male	56	Ulna	Air	900	30	254,66	246,10	242,07	93,09	0,31	4,28
458	Male	56	Ulna	Air	900	30	250,60	244,00	241,90	92,13	0,10	3,97
459	Male	87	Radius	Air	400	30	57,14	67,75	62,62	23,54	-0,30	3,29
460	Male	87	Radius	Air	400	30	67,78	77,76	68,57	22,24	-0,24	3,41
461	Male	87	Radius	Air	400	30	68,22	79,82	72,42	27,32	0,55	3,60
462	Male	87	Radius	Air	400	30	56,67	62,52	55,25	16,33	0,16	3,38
463	Male	87	Radius	Air	450	10	72,93	81,02	74,95	27,97	0,13	3,79
464	Male	87	Radius	Air	400	30	49,78	57,98	52,65	21,90	-0,15	3,82
465	Male	87	Radius	Air	400	30	53,33	62,44	56,35	23,33	-0,22	3,29
466	Male	87	Radius	Air	450	10	67,94	76,81	71,09	26,97	-0,05	3,14
467	Male	87	Radius	Air	450	10	69,94	78,32	72,44	25,97	-0,77	2,94
468	Male	87	Radius	Air	450	10	67,94	76,43	71,17	27,97	0,23	3,94
469	Male	87	Radius	Air	450	10	66,94	75,32	70,08	29,97	-0,11	3,18
470	Male	87	Radius	Air	450	20	79,93	88,13	81,85	27,97	-0,48	3,14

Table s9. Raw data of the learning set (12/20)

ID nr	Sex	Age	Skeletal element	Medium	Temp. (°C)	Time (min.)	R	G	B	L*	A*	B*
471	Male	87	Radius	Air	450	20	60,94	68,94	63,13	23,98	-0,01	3,34
472	Male	87	Radius	Air	450	20	76,93	84,54	79,84	25,97	-0,23	3,26
473	Male	87	Radius	Air	450	20	65,94	73,34	69,03	26,97	0,37	3,81
474	Male	87	Radius	Air	450	20	72,93	80,93	76,03	21,98	-0,43	3,88
475	Male	87	Radius	Air	450	20	79,93	87,49	82,76	27,97	0,62	3,36
476	Male	87	Radius	Fat	400	30	63,45	72,34	66,00	22,42	-0,48	3,14
477	Male	87	Radius	Fat	400	30	60,48	67,64	60,88	24,65	0,14	4,07
478	Male	87	Radius	Fat	400	30	69,21	77,72	70,61	27,32	0,64	3,88
479	Male	87	Radius	Fat	400	30	63,30	72,75	67,24	30,72	-0,15	3,18
480	Male	87	Radius	Fat	400	30	60,94	69,92	62,81	24,98	0,88	3,57
481	Male	87	Radius	Fat	450	10	63,94	72,24	66,61	21,98	1,88	4,89
482	Male	87	Radius	Fat	400	30	67,94	74,92	68,81	22,98	0,25	3,38
483	Male	87	Radius	Fat	450	10	68,94	77,44	72,11	21,98	0,77	3,29
484	Male	87	Radius	Fat	450	10	69,94	78,00	71,86	23,98	2,77	4,88
485	Male	87	Radius	Fat	450	10	64,94	73,22	66,96	22,98	3,33	6,14
486	Male	87	Radius	Fat	450	10	69,94	78,33	71,92	21,98	2,23	6,90
487	Male	87	Radius	Fat	450	10	65,94	74,21	68,40	24,98	0,99	6,64
488	Male	87	Radius	Fat	450	20	59,95	68,72	62,52	20,98	2,37	7,34
489	Male	87	Radius	Fat	450	20	56,95	65,89	60,00	18,98	0,79	4,37
490	Male	87	Radius	Fat	450	20	60,94	69,74	63,16	23,98	0,99	3,20
491	Male	87	Radius	Fat	450	20	58,95	67,61	61,39	23,98	3,33	6,90
492	Male	87	Radius	Fat	450	20	52,52	61,89	56,29	20,98	0,57	4,05
493	Male	87	Radius	Fat	450	20	61,94	69,95	64,65	24,98	2,31	5,44
494	Male	87	Ulna	Air	400	30	66,62	74,45	66,57	23,77	-0,22	3,51
495	Male	87	Ulna	Air	400	30	65,00	73,17	68,26	24,43	-0,15	3,55
496	Male	87	Ulna	Air	400	30	61,54	68,42	63,28	19,59	-0,13	3,16
497	Male	87	Ulna	Air	400	30	44,55	51,64	46,64	14,23	0,18	3,73
498	Male	87	Ulna	Air	450	10	67,94	76,09	70,30	23,98	0,54	3,88
499	Male	87	Ulna	Air	400	30	54,51	63,16	56,98	22,69	-0,15	3,65
500	Male	87	Ulna	Air	400	30	44,20	51,65	46,43	19,45	-0,33	3,33
501	Male	87	Ulna	Air	450	10	73,93	82,46	76,72	24,98	0,00	3,27
502	Male	87	Ulna	Air	450	10	73,93	82,77	76,70	23,98	-0,54	3,36
503	Male	87	Ulna	Air	450	10	67,94	76,77	70,83	28,97	-0,43	3,35
504	Male	87	Ulna	Air	450	10	70,93	79,72	74,24	25,97	0,31	3,73
505	Male	87	Ulna	Fat	400	30	67,12	75,72	70,52	22,31	0,55	3,73
506	Male	87	Ulna	Fat	400	30	64,22	73,17	67,41	28,20	-0,05	3,80
507	Male	87	Ulna	Fat	400	30	60,35	68,74	62,61	19,66	-0,33	2,94
508	Male	87	Ulna	Fat	400	30	70,39	78,23	71,82	31,39	0,20	3,80
509	Male	87	Ulna	Fat	400	30	65,94	73,92	69,81	26,97	-0,42	2,94
510	Male	87	Ulna	Fat	450	10	66,94	75,75	69,92	22,98	1,46	4,52
511	Male	87	Ulna	Fat	400	30	67,98	73,92	69,81	30,97	0,36	3,88
512	Male	87	Ulna	Fat	450	10	59,95	68,31	62,65	21,98	1,37	3,92
513	Male	87	Ulna	Fat	450	10	68,94	77,71	71,26	19,98	0,89	3,45

Table s9. Raw data of the learning set (13/20)

ID nr	Sex	Age	Skeletal element	Medium	Temp. (°C)	Time (min.)	R	G	B	L*	A*	B*
514	Male	87	Ulna	Fat	450	10	61,43	70,72	65,03	20,98	1,17	3,03
515	Male	87	Ulna	Fat	450	10	66,94	74,94	68,82	21,98	0,54	7,26
516	Male	87	Ulna	Fat	450	10	60,94	69,38	63,02	20,98	3,12	2,94
517	Male	87	Ulna	Fat	450	20	62,94	71,01	65,95	22,98	0,09	3,05
518	Male	87	Ulna	Fat	450	20	62,94	71,62	65,87	20,98	0,12	4,47
519	Male	87	Ulna	Fat	450	20	62,94	70,99	65,53	28,98	1,13	6,25
520	Male	87	Ulna	Fat	450	30	54,95	63,58	57,93	21,98	0,77	5,91
521	Male	87	Ulna	Fat	450	20	58,95	67,51	61,77	23,98	0,35	3,15
522	Male	87	Ulna	Fat	450	30	53,85	62,72	56,93	19,98	3,42	4,55
523	Female	65	Radius	Air	500	10	65,68	68,16	59,96	24,89	1,64	4,93
524	Female	65	Radius	Air	500	10	67,06	69,56	60,95	24,55	1,79	5,01
525	Female	65	Radius	Air	500	20	91,52	90,92	80,51	32,67	2,48	6,07
526	Female	65	Radius	Air	500	20	95,26	95,21	84,89	34,25	2,09	5,75
527	Female	65	Radius	Air	500	30	151,08	140,95	125,31	26,39	5,18	4,74
528	Female	65	Radius	Air	500	30	135,46	127,83	113,05	50,53	4,39	9,55
529	Female	65	Radius	Air	600	10	79,61	83,29	75,00	28,96	1,27	4,38
530	Female	65	Radius	Air	600	10	83,42	84,63	75,33	30,27	2,08	5,35
531	Female	65	Radius	Air	700	30	244,38	250,64	241,95	95,04	0,09	3,97
532	Female	65	Radius	Air	700	30	244,45	251,33	243,34	94,40	-0,01	3,60
533	Female	65	Radius	Air	600	20	77,42	81,48	73,35	29,80	1,12	4,08
534	Female	65	Radius	Air	600	20	101,07	102,26	92,71	38,84	2,14	5,54
535	Female	65	Radius	Air	600	30	104,29	105,90	96,79	38,07	1,95	5,08
536	Female	65	Radius	Air	600	30	104,64	105,51	95,71	38,86	2,11	5,60
537	Female	65	Radius	Air	700	10	131,06	135,58	127,48	51,85	0,97	4,06
538	Female	65	Radius	Air	700	10	137,77	139,75	130,13	55,04	1,58	5,47
539	Female	65	Radius	Air	700	20	214,04	220,95	213,01	83,75	0,07	3,80
540	Female	65	Radius	Air	700	20	214,25	221,19	212,96	83,97	-0,03	3,89
541	Female	65	Radius	Air	800	10	225,54	227,27	214,77	88,82	0,90	6,20
542	Female	65	Radius	Air	800	10	227,39	230,54	219,15	89,10	0,82	6,00
543	Female	65	Radius	Air	800	20	228,67	228,24	212,98	93,03	0,92	6,60
544	Female	65	Radius	Air	800	20	229,59	231,17	218,43	92,01	0,80	6,08
545	Female	65	Radius	Air	900	10	245,10	248,27	237,38	93,41	0,68	5,32
546	Female	65	Radius	Air	900	10	248,16	253,17	243,70	96,01	0,43	4,85
547	Female	65	Radius	Air	0	0	176,83	135,00	107,30	60,41	13,19	19,65
548	Female	65	Radius	Air	0	0	179,19	142,11	114,51	61,40	11,98	19,51
549	Female	65	Radius	Air	100	10	166,56	127,30	101,01	53,33	14,53	20,55
550	Female	65	Radius	Air	100	10	167,25	133,93	108,01	53,22	15,72	21,30
551	Female	65	Radius	Air	100	20	165,14	134,89	108,38	54,53	12,00	19,61
552	Female	65	Radius	Air	100	20	166,21	136,00	109,41	55,18	3,33	19,78
553	Female	65	Radius	Air	100	30	164,11	138,70	110,99	55,74	9,51	19,17
554	Female	65	Radius	Air	100	30	167,80	140,45	113,70	56,31	10,55	19,09
555	Female	65	Radius	Air	150	10	174,88	153,52	123,30	60,45	7,50	19,88
556	Female	65	Radius	Air	150	10	180,77	159,95	131,84	62,37	7,65	18,79

Table s9. Raw data of the learning set (14/20)

ID nr	Sex	Age	Skeletal element	Medium	Temp. (°C)	Time (min.)	R	G	B	L*	A*	B*
557	Female	65	Radius	Air	150	20	169,14	149,78	121,22	59,87	6,56	17,99
558	Female	65	Radius	Air	150	20	169,20	149,96	123,19	59,64	6,98	17,67
559	Female	65	Radius	Air	150	30	177,16	156,86	128,19	62,28	6,95	18,59
560	Female	65	Radius	Air	150	30	182,99	163,90	134,45	64,40	6,33	18,49
561	Female	65	Radius	Air	200	10	178,35	162,96	135,38	64,29	5,03	17,05
562	Female	65	Radius	Air	200	10	180,35	164,51	136,96	64,99	5,28	17,16
563	Female	65	Radius	Air	200	20	169,13	154,06	126,37	60,54	5,02	17,20
564	Female	65	Radius	Air	200	20	170,82	154,13	126,35	60,63	5,77	17,78
565	Female	65	Radius	Air	200	30	169,28	143,20	111,19	58,95	8,62	21,30
566	Female	65	Radius	Air	200	30	173,53	148,72	116,59	60,55	8,47	21,52
567	Female	65	Radius	Air	250	10	179,77	158,00	127,25	62,68	7,35	19,98
568	Female	65	Radius	Air	250	10	181,04	159,73	129,03	63,39	7,07	19,69
569	Female	65	Radius	Air	250	20	169,75	128,72	94,16	53,93	15,59	26,19
570	Female	65	Radius	Air	250	20	171,85	132,62	98,36	54,30	14,85	25,23
571	Female	65	Radius	Air	250	30	126,88	88,42	64,31	37,31	17,38	21,20
572	Female	65	Radius	Air	250	30	129,58	86,53	61,91	37,66	19,51	22,89
573	Female	65	Radius	Air	300	10	135,10	94,00	67,71	40,71	18,01	23,16
574	Female	65	Radius	Air	300	10	146,12	107,38	77,90	45,45	15,85	23,69
575	Female	65	Radius	Air	300	20	56,10	54,97	46,44	18,99	4,02	5,73
576	Female	65	Radius	Air	300	20	62,39	60,48	51,52	21,37	3,99	5,95
577	Female	65	Radius	Air	300	30	57,49	61,07	52,91	20,87	1,40	4,34
578	Female	65	Radius	Air	300	30	63,13	67,78	59,86	24,69	0,98	4,01
579	Female	65	Radius	Air	350	10	56,37	62,81	55,30	21,49	0,32	3,52
580	Female	65	Radius	Air	350	10	63,25	67,69	59,37	23,72	1,08	4,39
581	Female	65	Radius	Air	350	20	59,23	66,25	58,51	22,15	-0,03	3,52
582	Female	65	Radius	Air	350	20	61,39	69,81	62,76	24,44	-0,41	2,95
583	Female	65	Radius	Air	350	30	61,37	70,31	63,42	24,16	-0,58	2,71
584	Female	65	Radius	Air	350	30	61,87	70,93	64,05	24,42	-0,64	2,76
585	Female	65	Radius	Air	400	10	63,08	71,07	62,67	25,04	-0,40	3,27
586	Female	65	Radius	Air	400	10	63,38	71,83	64,67	25,61	-0,46	3,03
587	Female	65	Radius	Air	400	20	61,36	69,52	62,28	24,21	-0,36	3,09
588	Female	65	Radius	Air	400	20	68,90	76,38	68,41	26,92	-0,17	3,74
589	Female	65	Radius	Fat	100	10	168,70	140,67	114,13	56,95	11,48	19,79
590	Female	65	Radius	Fat	100	10	175,60	150,46	124,67	58,37	10,56	18,63
591	Female	65	Radius	Fat	100	20	157,47	130,17	105,85	49,84	13,27	18,91
592	Female	65	Radius	Fat	100	20	159,28	133,96	110,42	51,09	12,44	18,21
593	Female	65	Radius	Fat	100	30	164,96	141,41	114,04	56,58	8,87	18,74
594	Female	65	Radius	Fat	100	30	168,75	147,28	121,30	58,09	8,36	17,80
595	Female	65	Radius	Fat	150	10	162,74	139,78	115,97	54,84	9,70	17,08
596	Female	65	Radius	Fat	150	10	166,40	143,64	118,58	55,07	9,87	17,90
597	Female	65	Radius	Fat	150	20	168,75	147,28	121,30	67,31	5,90	17,96
598	Female	65	Radius	Fat	150	20	185,10	166,83	138,16	64,00	7,01	18,96
599	Female	65	Radius	Fat	150	30	181,05	161,76	133,52	63,47	6,76	18,08

Table s9. Raw data of the learning set (15/20)

ID nr	Sex	Age	Skeletal element	Medium	Temp. (°C)	Time (min.)	R	G	B	L*	A*	B*
600	Female	65	Radius	Fat	150	30	183,65	165,61	137,95	65,99	5,96	17,65
601	Female	65	Radius	Fat	200	10	176,73	158,15	131,37	61,81	6,92	17,51
602	Female	65	Radius	Fat	200	10	176,80	157,36	129,87	60,82	7,59	18,28
603	Female	65	Radius	Fat	200	20	182,10	167,17	141,94	64,53	5,88	16,14
604	Female	65	Radius	Fat	200	20	184,41	169,85	144,21	65,90	5,23	15,99
605	Female	65	Radius	Fat	200	30	172,64	155,92	130,08	61,04	6,11	16,55
606	Female	65	Radius	Fat	200	30	179,67	163,52	137,60	62,84	6,53	17,06
607	Female	65	Radius	Fat	250	10	162,48	146,85	121,78	57,11	6,02	16,22
608	Female	65	Radius	Fat	250	10	168,69	154,84	130,08	61,01	1,42	15,40
609	Female	65	Radius	Fat	250	20	179,95	159,49	129,61	62,29	6,71	18,95
610	Female	65	Radius	Fat	250	20	180,28	160,18	130,47	63,11	7,14	19,39
611	Female	65	Radius	Fat	250	30	171,11	132,48	99,53	54,68	14,47	24,39
612	Female	65	Radius	Fat	250	30	172,11	134,81	101,53	55,68	13,79	24,25
613	Female	65	Radius	Fat	300	10	176,66	158,00	130,58	60,30	7,45	18,38
614	Female	65	Radius	Fat	300	10	181,43	162,28	133,94	64,87	0,39	18,11
615	Female	65	Radius	Fat	300	20	122,56	91,22	69,84	37,57	14,35	18,38
616	Female	65	Radius	Fat	300	20	125,24	93,18	71,21	38,27	14,29	18,56
617	Female	65	Radius	Fat	300	30	89,03	75,42	60,13	29,75	8,40	12,29
618	Female	65	Radius	Fat	300	30	90,74	77,32	62,94	30,21	8,47	11,78
619	Female	65	Radius	Fat	350	10	178,34	147,20	114,25	59,39	10,99	22,61
620	Female	65	Radius	Fat	350	10	178,58	147,43	114,40	59,83	10,95	22,81
621	Female	65	Radius	Fat	350	20	57,21	64,63	57,51	22,29	0,08	3,22
622	Female	65	Radius	Fat	350	20	57,96	64,75	57,32	22,47	0,24	3,42
623	Female	65	Radius	Fat	350	30	58,58	65,35	57,92	22,30	0,31	3,51
624	Female	65	Radius	Fat	350	30	61,57	65,82	57,33	23,21	1,03	4,48
625	Female	65	Radius	Fat	400	10	57,05	61,97	54,06	21,26	0,93	4,06
626	Female	65	Radius	Fat	400	10	58,05	64,10	56,54	21,96	0,68	3,78
627	Female	65	Radius	Fat	400	20	51,69	60,34	53,87	19,67	-0,32	2,59
628	Female	65	Radius	Fat	400	20	58,45	66,75	56,67	22,85	-0,20	2,81
629	Female	65	Radius	Air	0	0	142,99	117,49	93,90	47,74	10,51	17,32
630	Female	65	Radius	Air	0	0	146,04	121,74	97,28	49,38	9,81	17,82
631	Female	65	Radius	Air	100	30	147,10	130,91	106,41	52,08	6,16	16,18
632	Female	65	Radius	Air	100	30	150,90	134,95	110,49	53,48	6,17	15,92
633	Female	65	Radius	Air	200	30	166,61	141,68	110,77	57,47	8,45	20,39
634	Female	65	Radius	Air	200	30	166,86	142,59	112,08	58,04	8,21	20,16
635	Female	65	Radius	Air	250	30	130,85	91,65	68,38	40,20	17,15	20,91
636	Female	65	Radius	Air	250	30	127,54	91,14	68,97	39,70	16,64	20,28
637	Female	65	Radius	Air	300	30	61,25	64,58	56,65	23,91	1,88	4,48
638	Female	65	Radius	Air	300	30	64,67	68,69	60,72	24,60	1,41	4,27
639	Female	65	Radius	Air	350	30	67,94	75,89	68,63	27,47	-0,26	3,11
640	Female	65	Radius	Air	350	30	68,45	76,01	68,70	27,41	0,05	3,26
641	Female	65	Radius	Air	400	20	73,57	80,64	72,91	29,48	0,04	3,61
642	Female	65	Radius	Air	400	20	73,78	80,36	72,36	30,14	0,12	3,78

Table s9. Raw data of the learning set (16/20)

ID nr	Sex	Age	Skeletal element	Medium	Temp. (°C)	Time (min.)	R	G	B	L*	A*	B*
643	Female	65	Radius	Air	450	30	102,31	99,71	88,86	38,95	3,27	6,78
644	Female	65	Radius	Air	450	30	122,76	115,30	101,90	45,32	4,88	9,03
645	Female	65	Radius	Fat	100	30	147,13	129,11	104,82	52,23	6,78	16,16
646	Female	65	Radius	Fat	100	30	148,13	129,55	105,19	51,53	7,29	16,40
647	Female	65	Radius	Fat	200	30	142,52	125,82	101,97	50,82	6,65	16,01
648	Female	65	Radius	Fat	200	30	145,37	125,58	101,25	54,80	7,09	16,43
649	Female	65	Radius	Fat	250	30	149,83	114,02	84,47	57,63	9,40	20,17
650	Female	65	Radius	Fat	250	30	152,23	114,55	84,31	47,69	14,92	23,33
651	Female	65	Radius	Fat	300	30	55,81	60,74	52,56	47,33	14,09	22,52
652	Female	65	Radius	Fat	300	30	57,69	61,10	52,46	21,31	1,37	4,71
653	Female	65	Radius	Fat	350	30	50,46	57,79	50,96	19,21	0,41	2,96
654	Female	65	Radius	Fat	350	30	52,05	59,62	52,69	21,33	0,97	3,92
655	Female	65	Radius	Fat	400	20	53,80	61,88	55,13	18,19	-0,05	3,88
656	Female	65	Radius	Fat	400	20	56,45	63,98	57,08	22,93	0,15	2,91
657	Female	65	Radius	Fat	450	30	132,78	129,64	118,14	51,03	3,01	6,86
658	Female	65	Radius	Fat	450	30	135,16	129,66	116,99	49,78	4,13	8,27
659	Female	65	Ulna	Air	500	10	55,24	62,34	54,72	20,91	0,16	3,53
660	Female	65	Ulna	Air	500	10	58,64	65,39	57,93	22,18	0,24	3,45
661	Female	65	Ulna	Air	500	20	77,94	75,18	64,96	27,61	3,56	6,80
662	Female	65	Ulna	Air	500	20	123,97	114,30	99,54	22,47	6,38	6,11
663	Female	65	Ulna	Air	500	30	121,86	114,03	100,02	44,48	4,77	9,30
664	Female	65	Ulna	Air	500	30	130,44	122,38	108,52	47,52	4,82	9,21
665	Female	65	Ulna	Air	600	10	84,21	85,07	75,40	31,33	2,24	5,53
666	Female	65	Ulna	Air	600	10	93,41	91,26	80,60	33,45	3,11	6,55
667	Female	65	Ulna	Air	700	30	238,72	248,91	243,22	93,80	-0,62	2,36
668	Female	65	Ulna	Air	700	30	227,57	234,55	227,14	88,43	0,12	3,43
669	Female	65	Ulna	Air	600	20	90,17	91,65	82,36	32,30	1,86	4,96
670	Female	65	Ulna	Air	600	20	90,51	90,39	80,55	32,36	2,52	5,81
671	Female	65	Ulna	Air	600	30	92,29	94,63	85,69	35,00	1,70	5,00
672	Female	65	Ulna	Air	600	30	94,27	95,77	86,38	35,26	1,96	5,30
673	Female	65	Ulna	Air	700	10	100,06	103,70	95,32	39,09	1,22	4,38
674	Female	65	Ulna	Air	700	10	112,62	115,09	105,87	43,35	1,61	5,19
675	Female	65	Ulna	Air	700	20	198,01	204,18	196,88	77,86	0,47	3,82
676	Female	65	Ulna	Air	700	20	217,04	225,53	219,13	86,29	-0,20	2,90
677	Female	65	Ulna	Air	800	10	247,33	253,68	245,04	95,82	0,02	4,05
678	Female	65	Ulna	Air	800	10	248,72	255,19	246,78	96,04	0,08	3,90
679	Female	65	Ulna	Air	800	20	250,51	256,78	248,40	97,06	0,12	4,05
680	Female	65	Ulna	Air	800	20	248,81	255,00	245,77	96,80	0,11	4,62
681	Female	65	Ulna	Air	900	10	247,03	252,88	244,35	95,24	0,23	3,95
682	Female	65	Ulna	Air	900	10	249,55	256,23	247,99	96,33	0,04	3,86
683	Female	65	Ulna	Air	0	0	167,53	136,74	114,00	54,53	14,08	18,44
684	Female	65	Ulna	Fat	0	0	164,06	129,00	104,91	53,15	14,37	19,41
685	Female	65	Ulna	Air	100	10	176,65	149,01	120,48	59,53	10,46	19,96

Table s9. Raw data of the learning set (17/20)

ID nr	Sex	Age	Skeletal element	Medium	Temp. (°C)	Time (min.)	R	G	B	L*	A*	B*
686	Female	65	Ulna	Air	100	10	182,75	156,61	129,33	61,58	10,33	19,56
687	Female	65	Ulna	Air	100	20	168,52	143,15	116,11	56,71	10,20	19,28
688	Female	65	Ulna	Air	100	20	173,74	149,66	123,96	60,14	9,20	17,80
689	Female	65	Ulna	Air	100	30	163,21	138,36	110,53	53,46	9,90	19,67
690	Female	65	Ulna	Air	100	30	167,41	144,69	119,38	56,51	9,53	18,37
691	Female	65	Ulna	Air	150	10	173,69	149,91	119,89	59,62	8,48	20,06
692	Female	65	Ulna	Air	150	10	178,78	156,65	128,04	60,90	8,23	19,31
693	Female	65	Ulna	Air	150	20	171,31	152,88	126,86	59,81	6,69	16,92
694	Female	65	Ulna	Air	150	20	173,06	155,07	128,87	60,50	6,79	17,60
695	Female	65	Ulna	Air	150	30	164,20	146,96	121,72	57,71	6,59	17,10
696	Female	65	Ulna	Air	150	30	164,43	148,75	123,59	58,32	5,87	16,51
697	Female	65	Ulna	Air	200	10	179,04	161,88	135,21	63,47	6,11	17,40
698	Female	65	Ulna	Air	200	10	179,68	164,12	137,78	64,41	5,38	16,50
699	Female	65	Ulna	Air	200	20	179,89	158,42	127,25	62,99	6,94	19,69
700	Female	65	Ulna	Air	200	20	183,56	162,45	131,82	64,81	6,36	18,73
701	Female	65	Ulna	Air	200	30	169,77	148,68	118,23	59,89	6,73	19,38
702	Female	65	Ulna	Air	200	30	172,56	151,55	121,44	60,50	6,96	19,43
703	Female	65	Ulna	Air	250	10	176,30	155,10	125,25	61,94	6,93	18,97
704	Female	65	Ulna	Air	250	10	180,30	160,00	130,58	64,06	6,62	18,62
705	Female	65	Ulna	Air	250	20	150,76	113,95	81,90	47,35	14,08	24,20
706	Female	65	Ulna	Air	250	20	153,33	113,98	85,02	46,25	15,56	22,61
707	Female	65	Ulna	Air	250	30	111,10	79,94	61,92	37,55	17,13	19,71
708	Female	65	Ulna	Air	250	30	119,91	84,78	64,83	35,84	16,75	18,64
709	Female	65	Ulna	Air	300	10	136,75	96,36	70,89	41,44	13,65	22,37
710	Female	65	Ulna	Air	300	10	137,39	97,04	70,12	42,78	17,27	23,58
711	Female	65	Ulna	Air	300	20	65,88	66,53	57,87	24,94	3,01	5,50
712	Female	65	Ulna	Air	300	20	70,40	67,49	58,07	24,29	3,93	6,16
713	Female	65	Ulna	Air	300	30	66,58	67,60	58,97	24,99	2,76	5,36
714	Female	65	Ulna	Air	300	30	66,98	67,31	58,85	24,03	2,78	5,09
715	Female	65	Ulna	Air	350	10	55,37	60,39	52,53	21,33	0,66	3,96
716	Female	65	Ulna	Air	350	10	56,91	62,29	54,30	20,62	0,90	4,00
717	Female	65	Ulna	Air	350	20	63,76	72,05	64,81	27,10	-0,23	3,26
718	Female	65	Ulna	Air	350	20	67,61	75,36	67,89	25,66	-0,43	3,06
719	Female	65	Ulna	Air	350	30	57,56	65,96	58,88	24,82	-0,48	2,91
720	Female	65	Ulna	Air	350	30	62,51	71,18	64,12	23,28	-0,41	2,97
721	Female	65	Ulna	Air	400	10	68,12	76,23	68,78	28,99	0,05	3,59
722	Female	65	Ulna	Air	400	10	72,14	79,09	71,35	27,44	-0,41	3,21
723	Female	65	Ulna	Air	400	20	64,13	71,46	63,68	27,70	-0,20	3,53
724	Female	65	Ulna	Air	400	20	69,26	76,71	68,86	25,70	-0,14	3,55
725	Female	65	Ulna	Fat	100	10	159,84	134,08	107,18	56,29	8,43	18,45
726	Female	65	Ulna	Fat	100	10	164,00	141,81	114,85	54,00	9,30	18,52
727	Female	65	Ulna	Fat	100	20	175,94	150,44	122,20	60,42	9,10	19,13
728	Female	65	Ulna	Fat	100	20	179,25	155,40	128,65	63,11	8,18	17,76

Table s9. Raw data of the learning set (18/20)

ID nr	Sex	Age	Skeletal element	Medium	Temp. (°C)	Time (min.)	R	G	B	L*	A*	B*
729	Female	65	Ulna	Fat	100	30	152,28	128,95	103,71	52,27	8,54	17,25
730	Female	65	Ulna	Fat	100	30	156,41	134,87	110,52	54,19	8,14	16,65
731	Female	65	Ulna	Fat	150	10	175,36	150,44	123,00	61,54	8,17	18,11
732	Female	65	Ulna	Fat	150	10	176,42	153,28	126,37	62,55	7,36	17,45
733	Female	65	Ulna	Fat	150	20	180,22	160,42	132,55	64,03	6,42	17,63
734	Female	65	Ulna	Fat	150	20	180,25	159,72	132,10	64,61	6,41	17,34
735	Female	65	Ulna	Fat	150	30	177,74	154,75	126,01	62,88	7,48	18,60
736	Female	65	Ulna	Fat	150	30	181,51	161,14	133,13	64,04	6,42	17,44
737	Female	65	Ulna	Fat	200	10	174,52	157,69	131,15	63,95	5,17	16,44
738	Female	65	Ulna	Fat	200	10	175,52	158,10	130,75	64,04	5,22	16,86
739	Female	65	Ulna	Fat	200	20	164,62	148,12	123,02	58,56	5,99	16,07
740	Female	65	Ulna	Fat	200	20	174,22	157,50	132,16	62,24	6,40	16,81
741	Female	65	Ulna	Fat	200	30	181,02	161,36	134,38	64,06	6,47	17,00
742	Female	65	Ulna	Fat	200	30	183,47	162,76	135,09	64,50	7,29	18,10
743	Female	65	Ulna	Fat	250	10	168,99	150,74	123,87	60,01	6,39	17,32
744	Female	65	Ulna	Fat	250	20	164,52	143,22	114,99	56,33	7,70	18,53
745	Female	65	Ulna	Fat	250	20	169,10	146,42	117,65	58,79	8,08	19,29
746	Female	65	Ulna	Fat	250	30	160,57	121,35	89,83	50,86	15,24	24,32
747	Female	65	Ulna	Fat	250	30	161,98	122,61	90,40	51,83	15,16	24,91
748	Female	65	Ulna	Fat	300	10	176,04	157,81	131,94	62,71	6,47	16,77
749	Female	65	Ulna	Fat	300	10	183,55	165,09	137,97	65,15	6,14	17,02
750	Female	65	Ulna	Fat	300	20	107,68	80,57	62,74	32,75	12,19	15,48
751	Female	65	Ulna	Fat	300	20	119,73	88,38	66,45	36,77	14,32	18,65
752	Female	65	Ulna	Fat	300	30	61,90	62,55	53,73	22,02	2,42	5,15
753	Female	65	Ulna	Fat	300	30	65,28	65,89	56,97	23,42	2,53	5,34
754	Female	65	Ulna	Fat	350	10	126,41	92,52	71,14	38,31	15,29	18,52
755	Female	65	Ulna	Fat	350	10	128,19	92,51	70,31	39,03	15,99	19,38
756	Female	65	Ulna	Fat	350	20	56,11	63,41	56,30	21,64	0,02	3,11
757	Female	65	Ulna	Fat	350	20	56,57	64,18	55,12	22,37	-0,05	3,09
758	Female	65	Ulna	Fat	350	30	49,99	57,19	49,90	18,55	-0,04	3,12
759	Female	65	Ulna	Fat	350	30	55,68	62,76	55,48	21,29	0,11	3,28
760	Female	65	Ulna	Fat	400	10	58,64	65,60	58,45	22,62	0,22	3,24
761	Female	65	Ulna	Fat	400	10	59,76	65,79	58,24	22,55	0,38	3,51
762	Female	65	Ulna	Fat	400	20	52,46	61,03	54,52	20,46	-0,38	2,52
763	Female	65	Ulna	Fat	400	20	55,27	63,78	57,22	21,72	-0,34	2,59
764	Female	65	Ulna	Fat	0	0	179,09	156,11	125,98	62,26	7,70	19,41
765	Female	65	Ulna	Fat	0	0	187,24	165,21	135,59	65,11	7,56	19,16
766	Female	65	Ulna	Air	100	30	174,76	153,33	124,47	61,04	7,35	18,76
767	Female	65	Ulna	Air	100	30	177,52	155,10	126,03	62,03	7,63	19,38
768	Female	65	Ulna	Air	200	30	180,75	145,82	112,49	60,47	11,91	23,03
769	Female	65	Ulna	Air	200	30	191,76	159,99	125,84	64,81	10,90	23,29
770	Female	65	Ulna	Air	250	30	129,65	90,44	67,45	38,61	17,43	20,46
771	Female	65	Ulna	Air	250	30	131,76	92,87	69,99	39,90	17,43	20,62

Table s9. Raw data of the learning set (19/20)

ID nr	Sex	Age	Skeletal element	Medium	Temp. (°C)	Time (min.)	R	G	B	L*	A*	B*
772	Female	65	Ulna	Air	300	30	61,48	63,04	54,40	22,44	2,31	5,11
773	Female	65	Ulna	Air	300	30	64,06	66,94	58,68	24,22	1,94	4,76
774	Female	65	Ulna	Air	350	30	65,53	73,83	66,57	26,89	-0,22	3,18
775	Female	65	Ulna	Air	350	30	65,57	73,45	65,97	26,41	-0,11	4,47
776	Female	65	Ulna	Air	400	20	58,37	66,41	59,37	23,44	-0,13	3,06
777	Female	65	Ulna	Air	400	20	64,06	71,70	63,98	25,36	-0,16	3,49
778	Female	65	Ulna	Air	450	30	103,28	100,34	89,26	38,98	3,48	7,03
779	Female	65	Ulna	Air	450	30	115,67	111,16	99,43	42,97	3,82	7,54
780	Female	65	Ulna	Fat	100	30	157,70	129,89	100,76	52,71	10,47	20,57
781	Female	65	Ulna	Fat	100	30	158,68	131,22	101,65	53,44	10,20	20,83
782	Female	65	Ulna	Fat	200	30	177,53	154,07	124,65	61,31	7,95	19,11
783	Female	65	Ulna	Fat	200	30	177,68	153,99	124,15	61,77	7,98	19,60
784	Female	65	Ulna	Fat	250	30	150,20	114,93	85,60	47,37	14,33	22,63
785	Female	65	Ulna	Fat	250	30	157,04	119,04	88,09	49,61	14,96	23,70
786	Female	65	Ulna	Fat	300	30	53,55	58,27	50,39	21,52	0,93	3,96
787	Female	65	Ulna	Fat	300	30	58,90	62,84	54,77	22,01	1,11	4,14
788	Female	65	Ulna	Fat	350	30	47,21	54,87	47,82	18,10	-0,06	3,04
789	Female	65	Ulna	Fat	350	30	51,25	58,99	52,00	19,78	-0,14	2,97
790	Female	65	Ulna	Fat	400	20	44,92	52,89	46,10	16,09	0,04	2,80
791	Female	65	Ulna	Fat	400	20	47,95	55,87	49,29	18,29	0,09	2,69
792	Female	65	Ulna	Fat	450	30	136,32	132,01	119,75	51,51	3,47	7,60
793	Female	65	Ulna	Fat	450	30	131,17	129,52	119,01	50,23	2,93	6,23
794	Female	54	Ulna	Fat	0	0	163,67	108,50	112,17	50,64	17,15	17,76
795	Female	54	Ulna	Air	0	0	160,17	123,00	102,34	49,97	15,29	21,74
796	Female	54	Radius	Fat	0	0	160,17	118,50	108,34	49,06	18,88	17,38
797	Female	54	Radius	Fat	0	0	156,67	117,16	98,34	48,05	16,98	21,61
798	Female	54	Ulna	Air	0	0	156,33	114,16	102,84	47,19	19,09	17,86
799	Female	54	Ulna	Fat	0	0	152,67	104,33	95,17	47,20	22,81	18,33
800	Female	54	Radius	Air	0	0	174,33	141,33	119,84	57,40	12,14	20,52
801	Female	54	Radius	Fat	0	0	168,50	118,00	113,84	53,88	13,48	21,11
802	Female	54	Ulna	Air	0	0	139,33	104,00	88,67	42,01	15,31	18,93
803	Female	54	Ulna	Air	0	0	148,50	111,50	97,34	45,42	16,06	18,85
804	Female	54	Radius	Fat	0	0	152,33	117,66	99,00	47,53	14,10	20,48
805	Female	54	Radius	Air	0	0	162,83	125,50	105,34	50,36	15,76	21,80
806	Female	54	Ulna	Fat	0	0	167,83	127,00	115,00	51,53	17,68	19,04
807	Female	54	Ulna	Air	0	0	165,83	131,16	109,34	45,19	13,48	21,72
808	Female	54	Radius	Fat	0	0	164,17	123,16	111,17	50,31	18,38	17,39
809	Female	54	Radius	Air	0	0	169,00	133,50	114,34	54,04	13,95	20,28
810	Female	54	Ulna	Fat	0	0	164,67	121,16	109,34	50,46	19,55	18,35
811	Female	54	Ulna	Air	0	0	158,67	121,83	109,50	49,76	15,81	16,46
812	Female	54	Radius	Fat	0	0	177,83	145,33	123,00	58,78	11,08	20,07
813	Female	54	Radius	Fat	0	0	183,50	145,83	124,84	59,01	14,46	21,55
814	Female	81	Humerus	Air	0	0	159,17	118,83	90,00	48,25	16,55	28,22

Table s9. Raw data of the learning set (20/20)

ID nr	Sex	Age	Skeletal element	Medium	Temp. (°C)	Time (min.)	R	G	B	L*	A*	B*
815	Female	81	Radius	Fat	0	0	149,00	115,16	91,84	46,35	13,23	23,14
816	Female	81	Ulna	Air	0	0	164,83	125,16	98,17	51,33	16,06	26,47
817	Female	81	Humerus	Fat	0	0	155,67	120,66	91,50	48,21	13,40	26,86
818	Female	81	Radius	Air	0	0	152,00	114,50	88,50	49,54	12,60	24,95
819	Female	81	Ulna	Fat	0	0	160,17	106,66	98,50	50,29	14,69	24,87
820	Female	81	Humerus	Air	0	0	152,83	112,33	84,67	49,11	13,24	25,57
821	Female	81	Radius	Fat	0	0	152,83	115,66	89,67	47,20	14,82	25,31
822	Female	81	Ulna	Air	0	0	153,33	118,83	93,84	47,31	13,64	24,13
823	Female	81	Humerus	Fat	0	0	154,33	118,16	94,50	46,60	13,88	22,91
824	Female	81	Radius	Air	0	0	151,67	116,83	87,67	41,66	14,82	26,80
825	Female	81	Ulna	Fat	0	0	154,00	119,16	95,00	48,29	13,56	23,86
826	Female	81	Humerus	Air	0	0	168,83	135,16	108,34	53,57	12,48	24,40
827	Female	81	Radius	Fat	0	0	152,67	118,00	91,50	47,52	15,25	25,37
828	Female	81	Ulna	Air	0	0	157,67	120,83	94,34	42,40	14,54	25,71
829	Female	81	Humerus	Fat	0	0	162,67	123,83	98,00	50,58	15,62	25,50
830	Female	81	Radius	Air	0	0	155,00	96,00	86,67	50,05	14,29	25,30
831	Female	81	Humerus	Fat	0	0	148,17	89,33	79,84	45,93	15,65	23,11
832	Female	81	Radius	Air	0	0	139,67	103,50	79,84	41,53	12,35	21,43
833	Female	81	Ulna	Fat	0	0	162,33	119,83	95,50	53,05	14,13	23,63

Table s10. Raw data of the test set (1/14)

ID nr	Device	Size	Medium	Temp. (°C)	Time (min.)	L*	B*
1	Scanner HP	4mm	-	0	-	76,085	20,19
2	Scanner HP	4mm	-	0	-	66	21,551
3	Scanner HP	4mm	-	0	-	68,849	22,413
4	Scanner HP	4mm	-	0	-	67,61	24,974
5	Scanner HP	4mm	-	0	-	64,593	23,58
6	Scanner HP	4mm	Air	100	10	77,706	22,219
7	Scanner HP	4mm	Air	100	10	68,217	23,277
8	Scanner HP	4mm	Air	100	10	69,079	19,182
9	Scanner HP	4mm	Air	100	10	67,412	23,1
10	Scanner HP	4mm	Air	100	10	74,112	23,073
11	Scanner HP	4mm	Air	100	20	73,775	21,635
12	Scanner HP	4mm	Air	100	20	66,766	21,969
13	Scanner HP	4mm	Air	100	20	68,101	20,613
14	Scanner HP	4mm	Air	100	20	72,023	20,685
15	Scanner HP	4mm	Air	100	20	71,368	21,878
16	Scanner HP	4mm	Air	100	30	73,287	20,535
17	Scanner HP	4mm	Air	100	30	75,525	21,681
18	Scanner HP	4mm	Air	100	30	71,007	21,653
19	Scanner HP	4mm	Air	100	30	71,29	20,526
20	Scanner HP	4mm	Air	100	30	64,513	22,445

Table s10. Raw data of the test set (2/14)

ID nr	Device	Size	Medium	Temp. (°C)	Time (min.)	L*	B*
21	Scanner HP	4mm	Fat	100	20	63,006	20,182
22	Scanner HP	4mm	Fat	100	20	61,425	21,652
23	Scanner HP	4mm	Fat	100	20	61,878	22,076
24	Scanner HP	4mm	Fat	100	20	58,8	23,055
25	Scanner HP	4mm	Fat	100	20	56,776	18,461
26	Scanner HP	4mm	Fat	100	30	58,422	22,756
27	Scanner HP	4mm	Fat	100	30	58,047	19,089
28	Scanner HP	4mm	Fat	100	30	60,859	21,901
29	Scanner HP	4mm	Fat	100	30	63,249	22,321
30	Scanner HP	4mm	Fat	100	30	63,563	20,031
31	Scanner HP	4mm	Air	250	10	72,414	20,03
32	Scanner HP	4mm	Air	250	10	68,275	19,198
33	Scanner HP	4mm	Air	250	10	76,588	19,065
34	Scanner HP	4mm	Air	250	10	74,772	19,655
35	Scanner HP	4mm	Air	250	10	80,621	19,585
36	Scanner HP	4mm	Air	250	20	72,29	23,836
37	Scanner HP	4mm	Air	250	20	65,63	28,426
38	Scanner HP	4mm	Air	250	20	64,638	25,937
39	Scanner HP	4mm	Air	250	20	67,047	27,494
40	Scanner HP	4mm	Air	250	20	74,663	22,208
41	Scanner HP	4mm	Air	250	30	50,487	28,065
42	Scanner HP	4mm	Air	250	30	59,745	29,75
43	Scanner HP	4mm	Air	250	30	49,892	24,582
44	Scanner HP	4mm	Air	250	30	58,066	28,582
45	Scanner HP	4mm	Air	250	30	53,032	26,843
46	Scanner HP	4mm	Fat	250	20	62,595	20,737
47	Scanner HP	4mm	Fat	250	20	57	20,392
48	Scanner HP	4mm	Fat	250	20	54,152	18,292
49	Scanner HP	4mm	Fat	250	20	62,832	20,475
50	Scanner HP	4mm	Fat	250	20	56,978	20,7
51	Scanner HP	4mm	Fat	250	30	59,879	21,26
52	Scanner HP	4mm	Fat	250	30	59,19	20,545
53	Scanner HP	4mm	Fat	250	30	62,395	18,56
54	Scanner HP	4mm	Fat	250	30	53,511	18,167
55	Scanner HP	4mm	Fat	250	30	53,851	19,726
56	Scanner HP	4mm	Air	300	10	65,943	27,76
57	Scanner HP	4mm	Air	300	10	64,362	28,878
58	Scanner HP	4mm	Air	300	10	58,058	30,254
59	Scanner HP	4mm	Air	300	10	58,153	28,038
60	Scanner HP	4mm	Air	300	10	58,637	27,483
61	Scanner HP	4mm	Air	300	20	29,432	4,544
62	Scanner HP	4mm	Air	300	20	29,691	6,834
63	Scanner HP	4mm	Air	300	20	25,396	4,835

Table s10. Raw data of the test set (3/14)

ID nr	Device	Size	Medium	Temp. (°C)	Time (min.)	L*	B*
64	Scanner HP	4mm	Air	300	20	31,022	7,798
65	Scanner HP	4mm	Air	300	20	31,379	11,716
66	Scanner HP	4mm	Air	300	30	28,95	8,386
67	Scanner HP	4mm	Air	300	30	28,752	9,618
68	Scanner HP	4mm	Air	300	30	30,088	11,381
69	Scanner HP	4mm	Air	300	30	33,766	11,316
70	Scanner HP	4mm	Air	300	30	30,63	8,871
71	Scanner HP	4mm	Fat	300	20	61	21,531
72	Scanner HP	4mm	Fat	300	20	52,405	20,846
73	Scanner HP	4mm	Fat	300	20	58,438	19,196
74	Scanner HP	4mm	Fat	300	20	59,46	22,638
75	Scanner HP	4mm	Fat	300	20	54,424	20,213
76	Scanner HP	4mm	Fat	300	30	56,088	21,671
77	Scanner HP	4mm	Fat	300	30	62,239	24,576
78	Scanner HP	4mm	Fat	300	30	54,597	24,762
79	Scanner HP	4mm	Fat	300	30	50,722	21,189
80	Scanner HP	4mm	Fat	300	30	64,181	24,561
81	Scanner HP	4mm	Air	450	10	19,548	-0,305
82	Scanner HP	4mm	Air	450	10	18	-0,752
83	Scanner HP	4mm	Air	450	10	18,783	-0,331
84	Scanner HP	4mm	Air	450	10	19,767	-0,588
85	Scanner HP	4mm	Air	450	10	20,908	-0,676
86	Scanner HP	4mm	Air	450	20	27,976	-0,991
87	Scanner HP	4mm	Air	450	20	29,804	-1,159
88	Scanner HP	4mm	Air	450	20	32,901	-0,127
89	Scanner HP	4mm	Air	450	20	29,291	-0,901
90	Scanner HP	4mm	Air	450	20	29,022	-0,91
91	Scanner HP	4mm	Air	450	30	46,388	9,25
92	Scanner HP	4mm	Air	450	30	26,969	2,116
93	Scanner HP	4mm	Air	450	30	35,997	4,665
94	Scanner HP	4mm	Air	450	30	35,538	-0,421
95	Scanner HP	4mm	Air	450	30	34,308	1,971
96	Scanner HP	4mm	Air	500	10	30,447	-0,534
97	Scanner HP	4mm	Air	500	10	20,845	-1,1
98	Scanner HP	4mm	Air	500	10	29,892	-1,262
99	Scanner HP	4mm	Air	500	10	21,972	-0,727
100	Scanner HP	4mm	Air	500	10	28,023	0,269
101	Scanner HP	4mm	Air	500	20	46,458	6,429
102	Scanner HP	4mm	Air	500	20	45,039	6,974
103	Scanner HP	4mm	Air	500	20	42,134	5,912
104	Scanner HP	4mm	Air	500	20	39,855	4,509
105	Scanner HP	4mm	Air	500	20	45,652	6,413
106	Scanner HP	4mm	Air	500	30	46,141	5,01
107	Scanner HP	4mm	Air	500	30	44,487	6,508

Table s10. Raw data of the test set (4/14)

ID nr	Device	Size	Medium	Temp. (°C)	Time (min.)	L*	B*
108	Scanner HP	4mm	Air	500	30	45,381	6,152
109	Scanner HP	4mm	Air	500	30	40,012	4,253
110	Scanner HP	4mm	Air	500	30	39,669	3,981
111	Scanner HP	4mm	Air	650	10	36,617	0,667
112	Scanner HP	4mm	Air	650	10	37,462	1,225
113	Scanner HP	4mm	Air	650	10	35,317	0,905
114	Scanner HP	4mm	Air	650	10	39,893	0,732
115	Scanner HP	4mm	Air	650	10	34,459	0,894
116	Scanner HP	4mm	Air	650	20	46,604	1,57
117	Scanner HP	4mm	Air	650	20	43,951	0,832
118	Scanner HP	4mm	Air	650	20	41,328	1,668
119	Scanner HP	4mm	Air	650	20	42,518	0,982
120	Scanner HP	4mm	Air	650	20	37,395	1,034
121	Scanner HP	4mm	Air	650	30	55,767	2,623
122	Scanner HP	4mm	Air	650	30	60,037	0,827
123	Scanner HP	4mm	Air	650	30	66,984	2,569
124	Scanner HP	4mm	Air	650	30	59,054	0,874
125	Scanner HP	4mm	Air	650	30	54,968	1,065
126	Scanner HP	4mm	Air	700	10	62,189	-0,457
127	Scanner HP	4mm	Air	700	10	56,836	-1,37
128	Scanner HP	4mm	Air	700	10	37,599	-0,329
129	Scanner HP	4mm	Air	700	10	53,305	-0,753
130	Scanner HP	4mm	Air	700	10	43,083	-0,921
131	Scanner HP	4mm	Air	700	20	94,656	-1,364
132	Scanner HP	4mm	Air	700	20	89,987	-0,648
133	Scanner HP	4mm	Air	700	20	95,432	-0,686
134	Scanner HP	4mm	Air	700	20	91,922	-1,553
135	Scanner HP	4mm	Air	700	20	90,143	-1,272
136	Scanner HP	4mm	Air	700	30	74,121	-0,36
137	Scanner HP	4mm	Air	700	30	95,229	-0,931
138	Scanner HP	4mm	Air	700	30	63,838	1,153
139	Scanner HP	4mm	Air	700	30	84,667	-0,903
140	Scanner HP	4mm	Air	700	30	93,877	-1,278
141	Scanner HP	4mm	Air	850	10	94,191	-0,692
142	Scanner HP	4mm	Air	850	10	98,437	-1,22
143	Scanner HP	4mm	Air	850	10	92,929	-0,223
144	Scanner HP	4mm	Air	850	10	98,573	-0,702
145	Scanner HP	4mm	Air	850	10	91,861	-0,3
146	Scanner HP	4mm	Air	850	20	99,17	-1,852
147	Scanner HP	4mm	Air	850	20	98,242	-1,216
148	Scanner HP	4mm	Air	850	20	99,5	-1,728
149	Scanner HP	4mm	Air	850	20	99,661	-2,3
150	Scanner HP	4mm	Air	850	20	98,362	-2,275
151	Scanner HP	4mm	Air	850	30	98,754	-1,435

Table s10. Raw data of the test set (5/14)

ID nr	Device	Size	Medium	Temp. (°C)	Time (min.)	L*	B*
152	Scanner HP	4mm	Air	850	30	99,104	-1,116
153	Scanner HP	4mm	Air	850	30	99,11	-1,239
154	Scanner HP	4mm	Air	850	30	98,544	-1,528
155	Scanner HP	4mm	Air	850	30	99,457	-1,825
156	Scanner HP	4mm	Air	100	50	63,403	19,081
157	Scanner HP	4mm	Air	100	50	65,047	17,288
158	Scanner HP	4mm	Air	100	50	53,424	18,497
159	Scanner HP	4mm	Air	100	50	63,554	18,894
160	Scanner HP	4mm	Air	100	50	74,143	18,96
161	Scanner HP	4mm	Air	250	50	55,486	22,952
162	Scanner HP	4mm	Air	250	50	68,408	24,978
163	Scanner HP	4mm	Air	250	50	55,768	24,226
164	Scanner HP	4mm	Air	250	50	59,38	27,101
165	Scanner HP	4mm	Air	250	50	62,744	25,992
166	Scanner HP	4mm	Air	300	50	28,646	9,655
167	Scanner HP	4mm	Air	300	50	28,738	-0,092
168	Scanner HP	4mm	Air	300	50	26,281	-0,435
169	Scanner HP	4mm	Air	300	50	25,381	-0,106
170	Scanner HP	4mm	Air	300	50	33,659	-0,646
171	Scanner HP	4mm	Air	350	50	29,42	-1,058
172	Scanner HP	4mm	Air	350	50	33,266	-1,285
173	Scanner HP	4mm	Air	350	50	40,203	-0,584
174	Scanner HP	4mm	Air	350	50	38,333	-0,484
175	Scanner HP	4mm	Air	350	50	36,704	-0,758
176	Scanner HP	4mm	Air	450	50	34,014	0,046
177	Scanner HP	4mm	Air	450	50	34,515	-0,431
178	Scanner HP	4mm	Air	450	50	39,656	4,711
179	Scanner HP	4mm	Air	450	50	40,09	0,829
180	Scanner HP	4mm	Air	450	50	39,63	-0,582
181	Scanner HP	4mm	Air	500	50	50,309	7,475
182	Scanner HP	4mm	Air	500	50	47,049	7,103
183	Scanner HP	4mm	Air	500	50	50,131	7,33
184	Scanner HP	4mm	Air	500	50	46,967	6,363
185	Scanner HP	4mm	Air	500	50	49,841	6,47
186	Scanner HP	4mm	Air	600	50	47,803	5,6
187	Scanner HP	4mm	Air	600	50	45,663	2,758
188	Scanner HP	4mm	Air	600	50	46,318	3,268
189	Scanner HP	4mm	Air	600	50	42,252	3,427
190	Scanner HP	4mm	Air	600	50	43,138	3,47
191	Scanner HP	4mm	Air	650	50	82,816	0,315
192	Scanner HP	4mm	Air	650	50	77,679	0,945
193	Scanner HP	4mm	Air	650	50	64,181	2,939
194	Scanner HP	4mm	Air	650	50	64,022	3,325
195	Scanner HP	4mm	Air	650	50	72,366	1,746

Table s10. Raw data of the test set (6/14)

ID nr	Device	Size	Medium	Temp. (°C)	Time (min.)	L*	B*
196	Scanner HP	4mm	Air	700	50	97,611	-1,951
197	Scanner HP	4mm	Air	700	50	98,74	-2,443
198	Scanner HP	4mm	Air	700	50	97,751	0,24
199	Scanner HP	4mm	Air	700	50	98,44	-0,956
200	Scanner HP	4mm	Air	700	50	99,089	-0,963
201	Scanner HP	4mm	Air	850	50	97,304	2,901
202	Scanner HP	4mm	Air	850	50	96,971	1,378
203	Scanner HP	4mm	Air	850	50	97,354	0,804
204	Scanner HP	4mm	Air	850	50	98,307	1,992
205	Scanner HP	4mm	Air	850	50	97,499	0,569
206	Scanner HP	40-80mm	Air	100	30	76,302	18,506
207	Scanner HP	40-80mm	Air	100	30	80,909	19,067
208	Scanner HP	40-80mm	Air	100	30	57,591	15,147
209	Scanner HP	40-80mm	Air	100	30	66,942	16,154
210	Scanner HP	40-80mm	Air	100	30	68,15	13,923
211	Scanner HP	40-80mm	Air	250	30	73,124	14,36
212	Scanner HP	40-80mm	Air	250	30	84,052	11,522
213	Scanner HP	40-80mm	Air	250	30	70,769	13,521
214	Scanner HP	40-80mm	Air	250	30	76,136	11,653
215	Scanner HP	40-80mm	Air	250	30	68,959	10,823
216	Scanner HP	40-80mm	Air	300	30	45,132	17,037
217	Scanner HP	40-80mm	Air	300	30	24,56	2,943
218	Scanner HP	40-80mm	Air	300	30	23,747	7,313
219	Scanner HP	40-80mm	Air	300	30	25,502	3,709
220	Scanner HP	40-80mm	Air	300	30	40,232	15,453
221	Scanner HP	40-80mm	Air	350	30	16,141	-1,739
222	Scanner HP	40-80mm	Air	350	30	19,637	-1,813
223	Scanner HP	40-80mm	Air	350	30	18,127	-1,776
224	Scanner HP	40-80mm	Air	350	30	15,506	-1,903
225	Scanner HP	40-80mm	Air	350	30	19,065	-1,679
226	Scanner HP	40-80mm	Air	450	30	27,093	-1,234
227	Scanner HP	40-80mm	Air	450	30	36,358	-0,409
228	Scanner HP	40-80mm	Air	450	30	40,298	1,238
229	Scanner HP	40-80mm	Air	450	30	40,675	1,635
230	Scanner HP	40-80mm	Air	450	30	37,716	0,233
231	Scanner HP	40-80mm	Air	500	30	51,533	4,945
232	Scanner HP	40-80mm	Air	500	30	55,231	4,165
233	Scanner HP	40-80mm	Air	500	30	51,726	5,662
234	Scanner HP	40-80mm	Air	500	30	51,871	6,205
235	Scanner HP	40-80mm	Air	500	30	50,697	6,453
236	Scanner HP	40-80mm	Air	600	30	55,898	0,576
237	Scanner HP	40-80mm	Air	600	30	53,162	2,674
238	Scanner HP	40-80mm	Air	600	30	54,68	3,098
239	Scanner HP	40-80mm	Air	600	30	52,411	2,354

Table s10. Raw data of the test set (7/14)

ID nr	Device	Size	Medium	Temp. (°C)	Time (min.)	L*	B*
240	Scanner HP	40-80mm	Air	600	30	53,993	1,734
241	Scanner HP	40-80mm	Air	650	30	81,605	-0,191
242	Scanner HP	40-80mm	Air	650	30	68,484	0,379
243	Scanner HP	40-80mm	Air	650	30	72,627	0,924
244	Scanner HP	40-80mm	Air	650	30	70,545	0,241
245	Scanner HP	40-80mm	Air	650	30	75,704	1,269
246	Scanner HP	40-80mm	Air	700	30	79,437	-1,581
247	Scanner HP	40-80mm	Air	700	30	78,434	0,045
248	Scanner HP	40-80mm	Air	700	30	70,919	-5,253
249	Scanner HP	40-80mm	Air	700	30	93,412	-0,173
250	Scanner HP	40-80mm	Air	700	30	81,749	-0,459
251	Scanner HP	40-80mm	Air	850	30	84	4,031
252	Scanner HP	40-80mm	Air	850	30	90,557	2,254
253	Scanner HP	40-80mm	Air	850	30	86,782	5,053
254	Scanner HP	40-80mm	Air	850	30	96,389	1,836
255	Scanner HP	40-80mm	Air	850	30	95,989	2,326
256	Scanner HP	4mm	Air	100	5	50,83	15,730
257	Scanner HP	4mm	Air	100	5	49,86	14,588
258	Scanner HP	4mm	Air	100	5	46,77	17,073
259	Scanner HP	4mm	Air	100	5	45,76	14,750
260	Scanner HP	4mm	Air	100	5	46,83	16,428
261	Scanner HP	4mm	Air	250	5	54,99	14,017
262	Scanner HP	4mm	Air	250	5	57,02	14,205
263	Scanner HP	4mm	Air	250	5	54,80	14,102
264	Scanner HP	4mm	Air	250	5	54,52	11,848
265	Scanner HP	4mm	Air	250	5	58,30	14,135
266	Scanner HP	4mm	Air	300	5	57,78	13,480
267	Scanner HP	4mm	Air	300	5	54,80	17,457
268	Scanner HP	4mm	Air	300	5	59,38	15,858
269	Scanner HP	4mm	Air	300	5	53,52	13,697
270	Scanner HP	4mm	Air	300	5	57,14	15,437
271	Scanner HP	4mm	Air	350	5	57,23	16,183
272	Scanner HP	4mm	Air	350	5	62,48	16,512
273	Scanner HP	4mm	Air	350	5	55,23	18,957
274	Scanner HP	4mm	Air	350	5	52,46	19,987
275	Scanner HP	4mm	Air	350	5	51,72	18,133
276	Scanner HP	4mm	Air	450	5	19,49	-3,457
277	Scanner HP	4mm	Air	450	5	24,85	0,513
278	Scanner HP	4mm	Air	450	5	24,76	-1,368
279	Scanner HP	4mm	Air	450	5	23,11	0,607
280	Scanner HP	4mm	Air	450	5	21,64	-1,883
281	Scanner HP	4mm	Air	500	5	16,15	-3,753
282	Scanner HP	4mm	Air	500	5	21,33	-3,432
283	Scanner HP	4mm	Air	500	5	22,36	-4,628

Table s10. Raw data of the test set (8/14)

ID nr	Device	Size	Medium	Temp. (°C)	Time (min.)	L*	B*
284	Scanner HP	4mm	Air	500	5	22,36	-4,292
285	Scanner HP	4mm	Air	500	5	19,19	-4,110
286	Scanner HP	4mm	Air	600	5	18,31	-3,908
287	Scanner HP	4mm	Air	600	5	19,72	-3,485
288	Scanner HP	4mm	Air	600	5	20,84	-4,207
289	Scanner HP	4mm	Air	600	5	23,49	-2,987
290	Scanner HP	4mm	Air	600	5	17,62	-3,530
291	Scanner HP	4mm	Air	700	5	32,42	-3,467
292	Scanner HP	4mm	Air	700	5	30,71	-2,685
293	Scanner HP	4mm	Air	700	5	29,49	-3,412
294	Scanner HP	4mm	Air	700	5	27,40	-2,598
295	Scanner HP	4mm	Air	700	5	26,98	-2,952
296	Scanner HP	4mm	Air	800	5	85,09	-3,420
297	Scanner HP	4mm	Air	800	5	78,08	-4,370
298	Scanner HP	4mm	Air	800	5	86,20	-4,782
299	Scanner HP	4mm	Air	800	5	84,74	-4,240
300	Scanner HP	4mm	Air	800	5	86,48	-3,808
301	Scanner HP	4mm	Air	900	5	93,92	-4,268
302	Scanner HP	4mm	Air	900	5	94,46	-4,010
303	Scanner HP	4mm	Air	900	5	94,48	-4,318
304	Scanner HP	4mm	Air	900	5	92,32	-4,123
305	Scanner HP	4mm	Air	900	5	93,05	-4,045
1	Nikon D700	4mm	-	0	-	91,624	16,620
2	Nikon D700	4mm	-	0	-	94,475	33,879
3	Nikon D700	4mm	-	0	-	88,27	27,475
4	Nikon D700	4mm	-	0	-	86,02	33,520
5	Nikon D700	4mm	-	0	-	87,758	35,660
6	Nikon D700	4mm	Air	100	10	89,404	14,253
7	Nikon D700	4mm	Air	100	10	89,6	18,467
8	Nikon D700	4mm	Air	100	10	80,937	20,067
9	Nikon D700	4mm	Air	100	10	85,123	22,323
10	Nikon D700	4mm	Air	100	10	88,32	15,660
11	Nikon D700	4mm	Air	100	20	88,772	20,564
12	Nikon D700	4mm	Air	100	20	89,331	23,750
13	Nikon D700	4mm	Air	100	20	89,309	20,944
14	Nikon D700	4mm	Air	100	20	91,778	16,566
15	Nikon D700	4mm	Air	100	20	91,469	19,015
16	Nikon D700	4mm	Air	100	30	92,455	14,934
17	Nikon D700	4mm	Air	100	30	90,818	16,511
18	Nikon D700	4mm	Air	100	30	91,26	20,591
19	Nikon D700	4mm	Air	100	30	92,965	15,705
20	Nikon D700	4mm	Air	100	30	91,502	21,177
21	Nikon D700	4mm	Fat	100	20	86,272	39,908
22	Nikon D700	4mm	Fat	100	20	81,948	44,937

Table s10. Raw data of the test set (9/14)

ID nr	Device	Size	Medium	Temp. (°C)	Time (min.)	L*	B*
23	Nikon D700	4mm	Fat	100	20	87,506	32,985
24	Nikon D700	4mm	Fat	100	20	83,149	47,538
25	Nikon D700	4mm	Fat	100	20	77,944	41,475
26	Nikon D700	4mm	Fat	100	30	79,178	38,899
27	Nikon D700	4mm	Fat	100	30	74,682	47,234
28	Nikon D700	4mm	Fat	100	30	82,121	32,065
29	Nikon D700	4mm	Fat	100	30	87,655	31,296
30	Nikon D700	4mm	Fat	100	30	83,347	36,106
31	Nikon D700	4mm	Air	250	10	93,474	13,978
32	Nikon D700	4mm	Air	250	10	91,888	17,453
33	Nikon D700	4mm	Air	250	10	93,928	10,873
34	Nikon D700	4mm	Air	250	10	93,408	13,377
35	Nikon D700	4mm	Air	250	10	94,083	12,607
36	Nikon D700	4mm	Air	250	20	92,669	17,582
37	Nikon D700	4mm	Air	250	20	89,206	32,598
38	Nikon D700	4mm	Air	250	20	88,765	27,192
39	Nikon D700	4mm	Air	250	20	89,522	27,807
40	Nikon D700	4mm	Air	250	20	93,146	16,078
41	Nikon D700	4mm	Air	250	30	77,358	56,114
42	Nikon D700	4mm	Air	250	30	86,041	43,211
43	Nikon D700	4mm	Air	250	30	77,637	43,525
44	Nikon D700	4mm	Air	250	30	83,486	48,088
45	Nikon D700	4mm	Air	250	30	78,738	53,198
46	Nikon D700	4mm	Fat	250	20	71,554	33,751
47	Nikon D700	4mm	Fat	250	20	72,222	38,988
48	Nikon D700	4mm	Fat	250	20	81,815	34,255
49	Nikon D700	4mm	Fat	250	20	84,185	27,236
50	Nikon D700	4mm	Fat	250	20	84,248	34,660
51	Nikon D700	4mm	Fat	250	30	86,015	30,985
52	Nikon D700	4mm	Fat	250	30	79,834	35,565
53	Nikon D700	4mm	Fat	250	30	77,168	37,688
54	Nikon D700	4mm	Fat	250	30	76,382	38,797
55	Nikon D700	4mm	Fat	250	30	72,785	36,643
56	Nikon D700	4mm	Air	300	10	89,413	31,269
57	Nikon D700	4mm	Air	300	10	88,212	38,060
58	Nikon D700	4mm	Air	300	10	85,297	45,222
59	Nikon D700	4mm	Air	300	10	81,329	44,665
60	Nikon D700	4mm	Air	300	10	82,876	46,525
61	Nikon D700	4mm	Air	300	20	37,457	16,196
62	Nikon D700	4mm	Air	300	20	40,28	11,953
63	Nikon D700	4mm	Air	300	20	38,483	16,153
64	Nikon D700	4mm	Air	300	20	41,875	24,797
65	Nikon D700	4mm	Air	300	20	48,322	57,512
66	Nikon D700	4mm	Air	300	30	44,331	19,061

Table s10. Raw data of the test set (10/14)

ID nr	Device	Size	Medium	Temp. (°C)	Time (min.)	L*	B*
67	Nikon D700	4mm	Air	300	30	40,591	24,673
68	Nikon D700	4mm	Air	300	30	44,756	32,485
69	Nikon D700	4mm	Air	300	30	46,582	23,416
70	Nikon D700	4mm	Air	300	30	37,693	21,741
71	Nikon D700	4mm	Fat	300	20	78,051	40,426
72	Nikon D700	4mm	Fat	300	20	66,445	46,055
73	Nikon D700	4mm	Fat	300	20	68,758	36,071
74	Nikon D700	4mm	Fat	300	20	84,147	35,352
75	Nikon D700	4mm	Fat	300	20	81,205	37,268
76	Nikon D700	4mm	Fat	300	30	75,809	38,824
77	Nikon D700	4mm	Fat	300	30	77,518	46,305
78	Nikon D700	4mm	Fat	300	30	71,63	49,429
79	Nikon D700	4mm	Fat	300	30	68,173	39,427
80	Nikon D700	4mm	Fat	300	30	75,81	37,051
81	Nikon D700	4mm	Air	450	10	22,682	-1,748
82	Nikon D700	4mm	Air	450	10	14,269	-1,019
83	Nikon D700	4mm	Air	450	10	20,331	1,365
84	Nikon D700	4mm	Air	450	10	16,064	-0,527
85	Nikon D700	4mm	Air	450	10	27,549	-1,635
86	Nikon D700	4mm	Air	450	20	37,398	-4,620
87	Nikon D700	4mm	Air	450	20	41,702	-3,943
88	Nikon D700	4mm	Air	450	20	39,034	-3,323
89	Nikon D700	4mm	Air	450	20	40,293	-3,976
90	Nikon D700	4mm	Air	450	20	37,568	-3,527
91	Nikon D700	4mm	Air	450	30	57,596	11,078
92	Nikon D700	4mm	Air	450	30	29,312	3,089
93	Nikon D700	4mm	Air	450	30	26,018	3,470
94	Nikon D700	4mm	Air	450	30	28,408	-3,373
95	Nikon D700	4mm	Air	450	30	22,729	-0,698
96	Nikon D700	4mm	Air	500	10	12,002	-2,701
97	Nikon D700	4mm	Air	500	10	12,622	-1,411
98	Nikon D700	4mm	Air	500	10	25,885	-3,235
99	Nikon D700	4mm	Air	500	10	41,973	1,665
100	Nikon D700	4mm	Air	500	10	23,078	-3,155
101	Nikon D700	4mm	Air	500	20	54,213	4,621
102	Nikon D700	4mm	Air	500	20	54,49	7,662
103	Nikon D700	4mm	Air	500	20	54,988	8,512
104	Nikon D700	4mm	Air	500	20	53,555	6,150
105	Nikon D700	4mm	Air	500	20	56,482	8,330
106	Nikon D700	4mm	Air	500	30	71,699	6,031
107	Nikon D700	4mm	Air	500	30	65,703	5,787
108	Nikon D700	4mm	Air	500	30	52,329	3,444
109	Nikon D700	4mm	Air	500	30	65,999	7,142
110	Nikon D700	4mm	Air	500	30	46,918	2,257

Table s10. Raw data of the test set (11/14)

ID nr	Device	Size	Medium	Temp. (°C)	Time (min.)	L*	B*
111	Nikon D700	4mm	Air	650	10	43,093	0,469
112	Nikon D700	4mm	Air	650	10	39,83	-0,376
113	Nikon D700	4mm	Air	650	10	29,353	0,880
114	Nikon D700	4mm	Air	650	10	36,185	0,368
115	Nikon D700	4mm	Air	650	10	32,622	1,739
116	Nikon D700	4mm	Air	650	20	43,818	1,658
117	Nikon D700	4mm	Air	650	20	46,971	0,111
118	Nikon D700	4mm	Air	650	20	45,802	-0,206
119	Nikon D700	4mm	Air	650	20	45,796	-0,625
120	Nikon D700	4mm	Air	650	20	46,294	0,047
121	Nikon D700	4mm	Air	650	30	79,693	1,135
122	Nikon D700	4mm	Air	650	30	73,837	-1,181
123	Nikon D700	4mm	Air	650	30	75,617	-1,546
124	Nikon D700	4mm	Air	650	30	63,696	-2,866
125	Nikon D700	4mm	Air	650	30	67,966	0,190
126	Nikon D700	4mm	Air	700	10	65,456	-4,646
127	Nikon D700	4mm	Air	700	10	51,947	-6,234
128	Nikon D700	4mm	Air	700	10	48,767	-4,242
129	Nikon D700	4mm	Air	700	10	70,429	-5,662
130	Nikon D700	4mm	Air	700	10	41,818	-4,482
131	Nikon D700	4mm	Air	700	20	95,499	-0,602
132	Nikon D700	4mm	Air	700	20	93,867	-1,581
133	Nikon D700	4mm	Air	700	20	92,002	-2,639
134	Nikon D700	4mm	Air	700	20	95,504	-0,529
135	Nikon D700	4mm	Air	700	20	95,446	-0,869
136	Nikon D700	4mm	Air	700	30	85,977	-2,297
137	Nikon D700	4mm	Air	700	30	93,051	-2,265
138	Nikon D700	4mm	Air	700	30	85,167	-0,344
139	Nikon D700	4mm	Air	700	30	96,204	-1,151
140	Nikon D700	4mm	Air	700	30	97,35	-0,469
141	Nikon D700	4mm	Air	850	10	96,797	-0,447
142	Nikon D700	4mm	Air	850	10	97,464	-0,387
143	Nikon D700	4mm	Air	850	10	94,553	-0,459
144	Nikon D700	4mm	Air	850	10	97,026	-0,266
145	Nikon D700	4mm	Air	850	10	94,576	-0,014
146	Nikon D700	4mm	Air	850	20	97,454	-0,534
147	Nikon D700	4mm	Air	850	20	97,542	-0,338
148	Nikon D700	4mm	Air	850	20	98,137	-0,340
149	Nikon D700	4mm	Air	850	20	98,226	-0,243
150	Nikon D700	4mm	Air	850	20	98,249	-0,157
151	Nikon D700	4mm	Air	850	30	96,103	-0,263
152	Nikon D700	4mm	Air	850	30	96,988	-0,255
153	Nikon D700	4mm	Air	850	30	97,599	-0,190
154	Nikon D700	4mm	Air	850	30	98,214	-0,317

Table s10. Raw data of the test set (12/14)

ID nr	Device	Size	Medium	Temp. (°C)	Time (min.)	L*	B*
155	Nikon D700	4mm	Air	850	30	98,407	-0,254
156	Nikon D700	4mm	Air	100	50	82,711	21,822
157	Nikon D700	4mm	Air	100	50	80,792	21,651
158	Nikon D700	4mm	Air	100	50	67,5	30,426
159	Nikon D700	4mm	Air	100	50	69,858	26,731
160	Nikon D700	4mm	Air	100	50	87,89	16,508
161	Nikon D700	4mm	Air	250	50	69,741	42,252
162	Nikon D700	4mm	Air	250	50	86,54	28,346
163	Nikon D700	4mm	Air	250	50	72,246	45,556
164	Nikon D700	4mm	Air	250	50	71,542	47,405
165	Nikon D700	4mm	Air	250	50	68,334	41,477
166	Nikon D700	4mm	Air	300	50	21,733	15,622
167	Nikon D700	4mm	Air	300	50	11,039	-1,040
168	Nikon D700	4mm	Air	300	50	17,237	1,600
169	Nikon D700	4mm	Air	300	50	10,292	3,405
170	Nikon D700	4mm	Air	300	50	5,855	0,663
171	Nikon D700	4mm	Air	350	50	32,193	-3,964
172	Nikon D700	4mm	Air	350	50	35,048	-2,205
173	Nikon D700	4mm	Air	350	50	34,604	-1,244
174	Nikon D700	4mm	Air	350	50	22,957	-1,076
175	Nikon D700	4mm	Air	350	50	24,082	-0,251
176	Nikon D700	4mm	Air	450	50	36,668	3,796
177	Nikon D700	4mm	Air	450	50	23,163	-0,912
178	Nikon D700	4mm	Air	450	50	29,796	2,899
179	Nikon D700	4mm	Air	450	50	25,194	0,577
180	Nikon D700	4mm	Air	450	50	13,452	-0,272
181	Nikon D700	4mm	Air	500	50	42,813	9,529
182	Nikon D700	4mm	Air	500	50	39,821	8,864
183	Nikon D700	4mm	Air	500	50	46,735	9,547
184	Nikon D700	4mm	Air	500	50	31,014	9,647
185	Nikon D700	4mm	Air	500	50	45,633	8,810
186	Nikon D700	4mm	Air	600	50	54,174	5,094
187	Nikon D700	4mm	Air	600	50	41,101	-1,210
188	Nikon D700	4mm	Air	600	50	51,836	-1,085
189	Nikon D700	4mm	Air	600	50	58,411	2,700
190	Nikon D700	4mm	Air	600	50	48,683	-0,437
191	Nikon D700	4mm	Air	650	50	93,479	-0,225
192	Nikon D700	4mm	Air	650	50	92,017	0,031
193	Nikon D700	4mm	Air	650	50	86,57	0,405
194	Nikon D700	4mm	Air	650	50	86,343	0,474
195	Nikon D700	4mm	Air	650	50	91,568	0,107
196	Nikon D700	4mm	Air	700	50	96,811	-0,243
197	Nikon D700	4mm	Air	700	50	98,694	-0,207
198	Nikon D700	4mm	Air	700	50	98,545	-0,173

Table s10. Raw data of the test set (13/14)

ID nr	Device	Size	Medium	Temp. (°C)	Time (min.)	L*	B*
199	Nikon D700	4mm	Air	700	50	97,731	-0,159
200	Nikon D700	4mm	Air	700	50	97,37	-0,582
201	Nikon D700	4mm	Air	850	50	97,936	0,139
202	Nikon D700	4mm	Air	850	50	97,478	0,221
203	Nikon D700	4mm	Air	850	50	96,342	0,782
204	Nikon D700	4mm	Air	850	50	96,68	0,334
205	Nikon D700	4mm	Air	850	50	98,776	0,095
206	Nikon D700	40-80mm	Air	100	30	74,461	30,068
207	Nikon D700	40-80mm	Air	100	30	73,738	31,525
208	Nikon D700	40-80mm	Air	100	30	64,148	27,351
209	Nikon D700	40-80mm	Air	100	30	75,996	27,072
210	Nikon D700	40-80mm	Air	100	30	71,685	27,819
211	Nikon D700	40-80mm	Air	250	30	76,459	21,734
212	Nikon D700	40-80mm	Air	250	30	86,319	22,737
213	Nikon D700	40-80mm	Air	250	30	72,597	23,887
214	Nikon D700	40-80mm	Air	250	30	82,946	18,568
215	Nikon D700	40-80mm	Air	250	30	79,02	21,678
216	Nikon D700	40-80mm	Air	300	30	52,718	33,382
217	Nikon D700	40-80mm	Air	300	30	22,065	12,178
218	Nikon D700	40-80mm	Air	300	30	23,388	6,093
219	Nikon D700	40-80mm	Air	300	30	18,384	6,061
220	Nikon D700	40-80mm	Air	300	30	38,205	30,506
221	Nikon D700	40-80mm	Air	350	30	10,179	-1,720
222	Nikon D700	40-80mm	Air	350	30	5,582	-0,477
223	Nikon D700	40-80mm	Air	350	30	14,947	-1,175
224	Nikon D700	40-80mm	Air	350	30	15,88	-1,381
225	Nikon D700	40-80mm	Air	350	30	14,068	-2,973
226	Nikon D700	40-80mm	Air	450	30	28,713	-0,587
227	Nikon D700	40-80mm	Air	450	30	39,266	-0,599
228	Nikon D700	40-80mm	Air	450	30	33,915	0,522
229	Nikon D700	40-80mm	Air	450	30	32,244	1,856
230	Nikon D700	40-80mm	Air	450	30	36,528	2,579
231	Nikon D700	40-80mm	Air	500	30	52,539	1,723
232	Nikon D700	40-80mm	Air	500	30	59,178	4,697
233	Nikon D700	40-80mm	Air	500	30	46,532	8,306
234	Nikon D700	40-80mm	Air	500	30	47,309	5,287
235	Nikon D700	40-80mm	Air	500	30	52,434	10,213
236	Nikon D700	40-80mm	Air	600	30	57,002	0,006
237	Nikon D700	40-80mm	Air	600	30	48,145	2,510
238	Nikon D700	40-80mm	Air	600	30	52,969	3,533
239	Nikon D700	40-80mm	Air	600	30	48,474	1,565
240	Nikon D700	40-80mm	Air	600	30	53,871	-0,661
241	Nikon D700	40-80mm	Air	650	30	74,349	-1,645
242	Nikon D700	40-80mm	Air	650	30	72,068	-0,852

Table s10. Raw data of the test set (14/14)

ID nr	Device	Size	Medium	Temp. (°C)	Time (min.)	L*	B*
243	Nikon D700	40-80mm	Air	650	30	80,706	-0,816
244	Nikon D700	40-80mm	Air	650	30	72,425	-1,649
245	Nikon D700	40-80mm	Air	650	30	80,785	0,125
246	Nikon D700	40-80mm	Air	700	30	80,951	-3,028
247	Nikon D700	40-80mm	Air	700	30	78,385	-1,408
248	Nikon D700	40-80mm	Air	700	30	62,243	-11,000
249	Nikon D700	40-80mm	Air	700	30	90,694	0,209
250	Nikon D700	40-80mm	Air	700	30	77,085	-2,078
251	Nikon D700	40-80mm	Air	850	30	82	2,439
252	Nikon D700	40-80mm	Air	850	30	82,163	2,719
253	Nikon D700	40-80mm	Air	850	30	81,495	1,856
254	Nikon D700	40-80mm	Air	850	30	92,548	1,439
255	Nikon D700	40-80mm	Air	850	30	95,054	1,343
